# Pharmacy Homeless Outreach Engagement Non-medical Independent Prescribing Rx (PHOENIx) Community Pharmacy-Based Pilot Randomized Controlled Trial

**DOI:** 10.1007/s11524-025-00981-0

**Published:** 2025-06-09

**Authors:** Richard Lowrie, Vibhu Paudyal, Andrew McPherson, Helena Heath, Jane Moir, Natalie Allen, Nigel Barnes, Hugh Hill, Adnan Araf, Cian Lombard, Steven Ross, Sarah Tearne, Parbir Jagpal, Versha Cheed, Lee Middleton, Shabana Akhtar, George Provan, Jennifer Hislop, Andrea Williamson, Frances S. Mair

**Affiliations:** 1 Centre for Homelessness and Inclusion Health, School of Health in Social Science, University of Edinburgh, Edinburgh, UK; 2 Florence Nightingale Faculty of Nursing, Midwifery and Palliative Care, King’s College London, London, UK; 3 School of Pharmacy, College of Medical and Dental Sciences, Birmingham, UK; 4 University of Birmingham, Birmingham, UK; 5 Pharmacy Services, NHS Greater Glasgow and Clyde, Scotland, UK; 6 NHS Birmingham and Solihull Mental Health Foundations Trust, Birmingham, UK; 7 SIFA Fireside, Birmingham, UK; 8 Simon Community Scotland, Glasgow, UK; 9 Birmingham Clinical Trials Unit, University of Birmingham, Birmingham, UK; 10 NHS Healthcare Improvement, Edinburgh, UK; 11 Undergraduate Medical School, School of Medicine, Dentistry and Nursing, College of Medical, Veterinary and Life Sciences, University of Glasgow, Glasgow, UK; 12 General Practice and Primary Care, Institute of Health and Wellbeing, College of Medical, Veterinary and Life Sciences, University of Glasgow, Glasgow, UK

**Keywords:** Homelessness, Community pharmacy, Integrated care, Underserved populations, RCT, Severe and multiple disadvantage

## Abstract

Randomized controlled trials (RCTs) aiming to address the multiple health and social challenges of people experiencing homelessness (PEH) are lacking. Here we report the findings from a multicenter, open, pilot RCT. The intervention involved independent prescriber pharmacist from the National Health Service working on outreach in partnership with dedicated workers from Homeless Voluntary Charity or Social Enterprises (HVCSEs) (Pharmacist and third sector charity worker integrated Homeless Outreach Engagement Non-medical Independent prescriber Rx’-PHOENIx) in low threshold HVCSE venues or temporary accommodation addressing PEH participants’ health and wider needs through repeated outreach. The trial aimed to investigate whether sufficient numbers of participants could be recruited, retained, the intervention delivered as planned, and sufficient data collected to inform a subsequent definitive RCT. Clinical outcomes were also collected at follow-up (6 months). Participants were recruited from five community pharmacies and nearby venues in urban centers of Glasgow-Scotland and Birmingham-England, then randomized one-to-one into PHOENIx intervention in addition to usual care (UC) or UC only. A priori progression criteria were achieved: 55% of those assessed as eligible were recruited; at 6 months, 72% remained in the study, 91% had emergency department and mortality data available, and 72% completed questionnaire booklets. Fifty-three percent of participants received at least 50% of the planned PHOENIx intervention consultations (in-person or phone) at 6 months. Signs of improvement in clinical outcomes in the PHOENIx group included fewer ambulance call-outs, ED visits, and hospitalizations; higher outpatient attendances; and higher scores on self-reported health-related quality of life. A definitive RCT is merited.

**Supplementary Information:**

The online version contains supplementary material available at 10.1007/s11524-025-00981-0.

## Introduction

Homelessness refers to rooflessness (e.g., street dwelling), houselessness (e.g., living in temporary shelter), living in insecure housing (threatened with or “sofa surfing”), or inadequate housing [1]. Homelessness including street dwelling has been steadily rising in the UK since 2010 [2, 3] and other high-income countries in the West [4]. Globally, around 2% of the world’s population (approximately 150 million people) are known to be experiencing homeless [5].

Poor health status including severe mental health illnesses and substance use disorders is both a cause and consequence of homelessness [6–8]. Adverse childhood experiences including being in foster care and childhood poverty are correlated with homelessness in later life [8, 9]. People experiencing homelessness (PEH) face stigma and discrimination in care settings which can lead to exclusion from care. Undiagnosed and untreated health problems including drug overdose, heart failure, accidents, and suicides lead to high early mortality [10–14]. Other key causes including cardiovascular disease, respiratory disease, and cancer are also amenable to prevention [12]. Lack of tailored support in streets and temporary accommodations can worsen health status and can be re-traumatizing for PEH [10].

Homelessness, together with substance misuse and criminal justice involvement, constitutes the overlapping core triad of severe and multiple disadvantages, adding adverse synergy to health and social care outcomes [8]. Integrated care combining health, housing, and social services is key to mitigating the severe disadvantages they face. However, PEH find services “fragmented,” not suitable to their needs, and based on appointment systems that they cannot navigate [15].

Despite the deleterious nature of homelessness, with exception [16, 17] very little in terms of gold standard randomized controlled trials (RCTs) evaluating interventions to improve health and social outcomes for PEH have been undertaken. PHOENIx (Pharmacy Homeless Outreach Engagement Non-medical Independent prescribers Rx) intervention involves an outreach partnership between NHS Independent prescribing pharmacist and Homeless Voluntary, Charity, or Social Enterprises (HVCSEs) locating PEH and offering weekly consultations to support the management of health and social issues. HVCSE staff often have strong trusting relationships with PEH, providing advocacy, support for applications for settled housing, benefits and advice, and making provision of food and clothing during times of crises. However, HVCSE staff operate separately and cannot readily share records with statutory services like healthcare; therefore, opportunities are often missed to capitalize on “windows of opportunity” with PEH. Evidence from a recent pilot RCT focused on PEH with history of non-fatal street drug overdose suggests improved outcomes from PHOENIx, recruited and supported in temporary accommodation and the streets, in Glasgow, UK [17]. An underpinning principle of PHOENIx was that an integrated approach to health, social, and practical care for PEH is important to address wider determinants of health and mitigate PEH barriers for engagement in primary care [18]. Other principles for PHOENIx include outreach (when services go to PEH rather than wait for PEH to attend services) which is preferable as a means of engaging and providing continuity of care to PEH [18].

Increasing access to care is important for PEH; lack of access is a contributor to poor health outcomes. Community pharmacies are accessible places to engage with PEH due to a pre-existing relationship, often connecting with PEH who are prescribed treatments for their physical and mental health problems. Opioid substitution treatment is often dispensed daily by community pharmacies for PEH (most of whom have substance use) [19] and needle exchange services create an opportunity to build a therapeutic milieu. Needle exchange service in UK community pharmacies is offered at no cost to intravenous substance users aged ≥ 18. Service users can also access information on harm reduction approaches and safe injection practices through pharmacies and obtain referrals to other services as required [20].

Community pharmacies located in urban centers can be a few steps away from where PEH live and sleep, and can be accessed without the need for an appointment. However, currently the interaction between PEH and pharmacy staff is often transactional and wider opportunities to offer holistic interventions are often missed.

Assertive outreach to assess and comprehensively address multiple complex health and social care needs is under-utilized in mainstream health and care in the UK [21]. The PHOENIx intervention aims to create a partnership between NHS pharmacists and HVCSE workers to offer a one stop shop for health and social care. Given the lack of previous evidence of the feasibility of recruiting from and delivering PHOENIx in Community Pharmacies; recruiting and delivering PHOENIx to PEH with and without previous substance overdose; and delivering the PHOENIx intervention across the UK outwith Glasgow and Edinburgh, a multicenter pilot study was needed prior to conducting a definitive multicenter RCT.

The aim of this study was to undertake a pilot multicenter RCT to assess the feasibility of conducting a larger, definitive trial assessing PHOENIx for PEH in a community pharmacy setting. In particular, this study aimed to determine the randomization process and recruitment rate measured by the numbers invited to participate, numbers recruited at baseline, retention of those remaining in the trial at 3-month and 6-month follow-up, adherence to the intervention, and the extent of data related to emergency department visits and mortality data collected at 6 months.

## Methods

### Study Design and Setting

PHOENIx community pharmacy study is a randomized, multicenter, open, parallel group pilot RCT, with parallel economic and qualitative process evaluations. It was conducted across two cities in the UK (Glasgow and Birmingham) representing high rates of socio-economic deprivation [22].

Glasgow (Scotland’s largest city) with a population of 600,000 residents has by far the largest number of homeless applications than any other local authority in Scotland [23] and has one of the highest rates of drug-related deaths in Europe and higher than in North America per head of population [13]. Between one-third and one-half of deaths in PEH are from drug misuse and Glasgow has the highest homeless death rate of any Scottish city [13]. In response, specific guidance [23] encourages same day initiation of opioid substitution treatment (OST) if a person presents asking and needing OST because OST if dosed optimally has a protective effect against drug deaths. According to the latest census, the population of the metropolitan borough of Birmingham sits at well over one million citizens, with those of white ethnicity making up half the population [24]. Birmingham (England) is one of the UK’s largest cities, and 600 individuals become homeless every week [25].

Recruitment venues were community pharmacies of urban areas (two in Glasgow, three in Birmingham) where potentially eligible participants frequented. A study protocol has been published elsewhere [26]. The study built on our previous descriptive studies [14, 27, 28] and our qualitative studies incorporating the views of PEH [15, 18, 29] and stakeholders, including service commissioners, healthcare professionals, and public health bodies [30].

### Participants and Recruitment

Participants were adults (≥ 18 years) experiencing homelessness (living in temporary homeless accommodation, no fixed abode, or rough sleeping/street dwelling). Participants were excluded if they were living in accommodation with 24-h support, including in-house medical support; intoxicated or in the opinion of the researcher, posing a safety risk to others, and deemed lacking the capacity to consent. All participants attended one of the respective study community pharmacies. Community pharmacy staff identified potentially eligible participants known to staff and referred them to the study researchers based in community pharmacies. Patients who presented with a prescription indicating no fixed abode, temporary shelters (hostels) as their address of correspondence or specialist homelessness general practice (family/primary care physician) as their care providers were approached. Pharmacy staff also used their existing relationship with patients attending the pharmacy to establish eligibility in relation to homelessness. Recruited participants also recommended the study to PEH in their acquaintance. Study researchers explained the study to eligible participants in pharmacies and or other venues, and provided them with a patient information leaflet. Attendance at the pharmacy could be for any reason including obtaining prescribed or over-the-counter medicines, wound management, injection equipment provision, and/or advice on health and wellbeing. Participants recruited from other venues were also users of the recruiting community pharmacies, who were identified and referred by the HVSCE or pharmacy staff. Potential participants presenting out of hours were invited to make a visit during the recruitment window (9–5 Monday to Friday). Researchers also visited their temporary residence or street locations with prior agreement.

After obtaining written informed consent including reassurance on confidentiality and voluntary nature of participation, study researchers collected information on baseline socio-demographics, physical health, mental health, addiction, treatments and prescribing, quality of life, accommodation (if any), subjective and objective measures of health and wellbeing, blood results, criminal justice encounters, welfare benefits, and service utilization/registration. These data were also collected in 3- and 6-month assessments by the study researchers; at each time point, the intention was to collect data in person and to supplement this with data extracted manually from each participant’s clinical records. Participants were offered a £10 shopping voucher on completion of each assessment.

### Randomization and Blinding

After baseline data were collected and in the presence of participants, researchers telephoned an interactive automated randomization number set up by Birmingham Clinical Trials Unit (BCTU). Participants were randomized at the level of the individual in a 1:1 ratio to either intervention group (IG) or usual care group (UG). The randomization list was generated by a statistician at BCTU and was stratified by recruiting city (Glasgow or Birmingham), using permuted blocks of varying lengths. It was not possible to blind participants or intervention staff owing to the nature of the intervention. Moreover, researchers could not be blinded because they provided the participant with their allocation to PHOENIx or UC and the same researchers followed up participants at 3 and 6 months.

Community pharmacy staff also supported the study by providing a private consulting space for baseline assessment, follow-up, and intervention delivery. The intervention team comprised an NHS employee pharmacist and HVCSE worker who were peripatetic (to maximize repeated engagement with participants who were itinerant), not community pharmacy contractor employees. The research team aimed to follow-up participants at 3 and 6 monthly intervals. Study methodology detailing proposed outcomes for the pilot RCT has been published [26].

### Intervention

Previous studies have described the PHOENIx model of care in other settings [14, 27, 28, 31, 32] with evaluations suggesting high levels of engagement from PEH [18, 33]. Briefly, it involved weekly visits by the PHOENIx team, offering support in terms of health and social needs. Taking approximately 1 h, in person (occasionally by phone), the PHOENIx team worked as per participant priorities, and assessed, treated (including prescribing medications), and referred to other health and social care teams. Health support included immediate prescribing for all presenting health needs falling within the pharmacist’s sphere of competence. One exception to this was in NHS Greater Glasgow and Clyde where the local Alcohol and Drug Recovery Service did not permit pharmacists (including PHOENIx pharmacists) other than those employed directly by the Alcohol and Drug Recovery Service, to initiate or continue treatments for opioid or other substance use. PHOENIx teams also offered support for participants to move into safer/better accommodation, practical and social support, e.g., applications for welfare benefits, essential goods, e.g., kettles, food, sleeping bags. In essence, the PHOENIx intervention is a health and social care collaboration, working on the premise that assertive outreach is the best means to engage and re-engage PEH with complex and enduring health and social care problems. The health element of the intervention is provided by NHS pharmacists who have undergone post-registration training as non-medical independent prescribers, and the social support role is carried out by an experienced worker from HVCSEs who helps with housing, benefits, employment, training, and leisure activities through social prescribing. The intervention team aimed to offer participants weekly contacts, and to support and tackle issues that the participants deemed of the highest priority at the time to them. Weekly meeting dates and venues where possible were agreed in advance between IG participants and the intervention team. However, the team utilized principles of assertive outreach through pro-active follow-up including street outreach and door knocking (temporary accommodations) to minimize participant barriers to making scheduled appointments and maximize engagement.

Trial participation did not alter usual care in either group as they continued to seek and obtain treatment or help as usual. As described in our study protocol [26], the researchers (and intervention team if on site) were able to signpost UG participants to relevant services where urgent healthcare needs were identified, e.g., leg ulcers needing urgent treatment.

### Outcomes

The main outcomes were feasibility progression criteria: red, amber, and green as described in Table 1. These related to (a) recruitment—proportion of PEH (as assessed by the researchers) meeting eligibility criteria and agreeing to participate; (b) retention—proportion of participants remaining in the study at 6 months; (c) intervention adherence—proportion of participants attending > 50% of intervention visits as planned; and (d) collection of outcomes data—proportion of participants with emergency department visits and mortality data available at 6 months and proportion of patients with questionnaire booklets completed at 6 months. Secondary outcomes are described in the published protocol (ISRCTN88146804) [26] which included a wide range of clinical, quality of life [34], addiction, and social outcomes. Follow-up outcomes were collected as close to the proposed timescales as possible.

**Table 1:** PHOENIx feasibility targets and achievements

	Red (discuss with Oversight Committee and consider substantial changes before proceeding to the definitive trial)	Amber (discuss with Oversight Committee strategies for improvement and consider changes to processes before deciding whether to proceed to full trial)	Green(go ahead)	Observed percentage (*N* %; 95% CI)
Recruitment				
Proportion of PEH (as assessed by the researchers) meeting eligibility criteria and agreeing to participate	< 40%	40–50%	> 50%	100/183 55% (0.47, 0.62)
Retention				
Proportion of participants remaining in the study at 6 months	< 50%	50–60%	> 60%	72/100 72% (0.63, 0.81)
Intervention adherence				
Proportion of participants attending > 50% of intervention visits as planned* (flexible schedule agreed at consultation)	< 50%	50–60%	> 60%	26/49 53% (0.39, 0.67)
Outcome data				
Proportion of participants with emergency department visits and mortality data available at 6 months	< 60%	60–70%	> 70%	91/100 91% (0.85, 0.97)
Proportion of patients with questionnaire booklets completed at 6 months	< 50%	50–60%	> 60%	72/100 72% (0.63, 0.81)

*At least eight visits over 4 months of the intervention period

### Sample Size and Statistical Methods

Based on previous work [32] and guidance on sample sizes for pilot studies [35], we aimed to recruit 30–50 patients per arm because the focus is on estimating the parameters for the full study, rather than formal testing of hypotheses. If the total number of eligible persons was 200, this would allow measurement of the recruitment rate with a 95% confidence interval width of approximately 14%. We estimated that if 70% of those recruited were followed up in terms of measuring ED visits, this would allow measurement of the rate with 95% confidence interval width of approximately 18%.

Feasibility measures were summarized using proportions, along with 95% confidence intervals (based on a normal approximation method for one sample proportions). We summarized participant characteristics and outcomes by applying frequencies as counts and percentages for categorical data, and depending on the distribution of continuous data, means and standard deviations (SD), or medians and intraquartile ranges (IQR). Given the pilot nature of the trial, no hypothesis testing was performed. The primary comparison groups were PHOENIx and UC.

### Patient and Public Involvement (PPI)

PPI is separately reported [36]. In summary, we undertook a series of patient and public involvement (PPI) activities throughout the life cycle of this research including input from a Lived Experience Advisory Panel and input from HVCSE staff working in terms of co-applicants, collaborators, and key members of the Trial Management Group. A HVCSE staff member was also a member of the Trial Oversight Committee.

## Results

### Primary Outcomes

#### Recruitment (Protocol Target: > 50% of PEH Assessed as Eligible Agreeing to Participate)

**Fig. 1:**
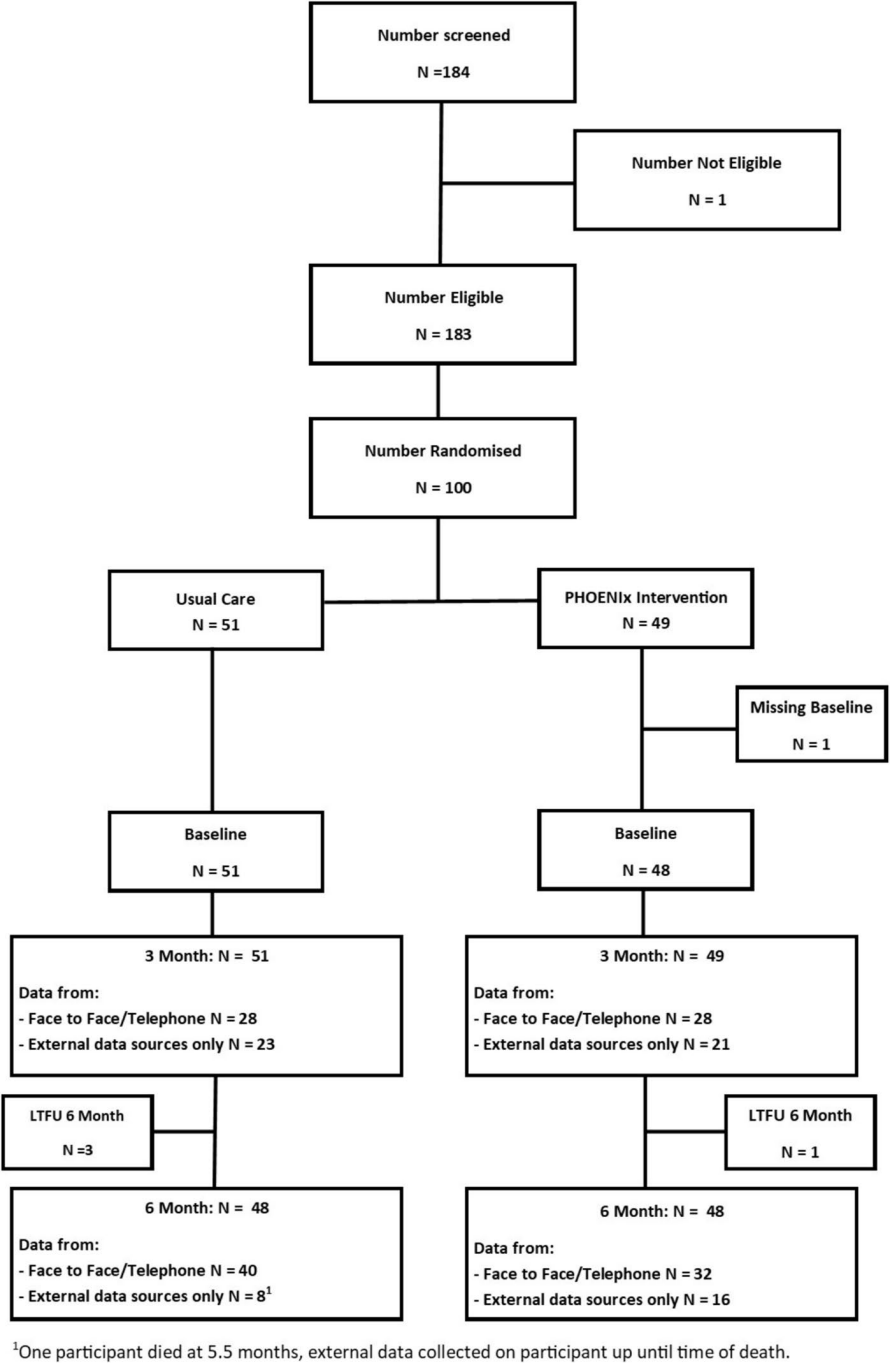
Consolidated standards of reporting trials flow diagram

The consort diagram (Fig. 1) shows recruitment, retention, and follow-up figures from the study. A total of 184 participants were screened and 183 (99.5%) were eligible from which the target 100 (55%, 95% CI (0.47, 0.62)) participants were recruited into the trial from five pharmacies (Supplementary material 1) between 17 th Nov 2022–16 th March 2023. These included 49 in PHOENIx and 51 in UC.

Baseline characteristics are separately reported (currently under review). In summary, participants were characterized by a high burden of poor mental health, substance use, and social disadvantages. Thirty-eight percent were registered with a specialist Homeless Health GP service and the remaining majority (bar one participant who had unknown GP registration information) were registered with mainstream GP practices. Fifty-nine percent had children. Sixty-two percent were living in a hostel, and 9% sleeping rough. Seventy-five percent were prescribed OST. Seventy-seven percent of participants had a mental health diagnosis (depression, anxiety, self-harm, post-traumatic stress disorder, and schizophrenia being the most common) and 38% were receiving treatment. Eighty-five percent had at least one chronic physical health problem (respiratory, wounds, blood borne viruses, pain, and skin conditions among the most common) with 44% receiving any kind of prescribed medicine for physical health conditions.

#### Participant Retention (Protocol Target > 60% Remaining in the Study at 6 Months)

Seventy-two (72%; 95% CI (0.63, 0.81)) participants were remaining in the study at 6 months, this is based on participants completing the follow-up questionnaire with the study researcher either face-to-face or telephone. Seventy-two (72%; 95% CI (0.63, 0.81)) participants had questionnaire booklets completed at 6 months. At 3 months, 56% of questionnaire booklets were completed (Table 1).

#### Intervention Adherence (Protocol Target: > 60% of Participants Attending > 50% of Intervention Visits as Planned)

Twenty-six (53%) participants had over half of the planned weekly contacts with the PHOENIx team (at least eight visits over 4 months of the intervention period) (Table 1). Forty-two (86%) participants had at least one contact with the intervention team over 6 months. Median face to face consultations over 6 months were 6.0 [IQR 1.0, 18.0] with a maximum of 64. Median telephone consultations over 6 months were 3.0 [IQR 0, 14.0] with a maximum of 46 (Supplementary material 2). Twenty-six participants, 53% (0.39–0.67), attended over half of the intervention visits as planned (Table 1).

#### Outcome Data Collection (Protocol Target: > 70% of Participants with Emergency Department and Mortality Data Available at 6 Months)

Proportion of participants with emergency department visits and mortality data available at 6 months was 91 (91%; 95% CI (0.85, 0.97)) (Table 1).

### Clinical Outcomes

Thirteen (31%) and 18 (40%) UC participants had at least one ED visit at 3- and 6-month follow-up respectively compared to 11 (22%) at baseline. In the UC, 4% of these visits related to drug overdose at baseline and none at the 6-month follow-up. In the IG, twelve (28%) participants at 3 months, and 12 (29%) partiticipants at 6 months had at least one ED attendance, compared to 14 (29%) at the baseline. In the IG, 6% visits in the baseline related to drug overdose compared to none (0%) in the 6-month follow-up (Supplementary material 3). Mortality data was collected with one death recorded in the intervention arm during the follow-up period (Fig. 1).

Hospitalization data was successfully obtained for all participants at baseline, 3, and 6 months. Six (12%) participants in UC had at least one hospital admissions in the 3-month period before the date of baseline data collection compared to 3 (7%) in the IG. This increased to 17 (36%) and 16 (34%) in 3 months; and 18 (40%) and 13 (29%) in the UC and IG respectively (Table 2). Causes of ED and hospital admissions are presented in Supplementary material 3.

**Table 2:** Clinical outcomes

		Usual care			PHOENIx intervention		
		Baseline (*n* = 51)	0–3 months (*n* = 51)	3–6 months (*n* = 48)	Baseline (*n* = 48)	0–3 months (*n* = 49)	3–6 months (*n* = 48)
Number of emergency department visits							
	Median (IQR)	0 [0–0]	0 [0–1]	0 [0–1]	0 [0–1.0]	0 [0–1]	0 [0–1]
	Min–max	0–7.0	0–4	0–4	0–3.0	0–5	0–6
	Missing	2	8	3	1	6	2
0	*N* (%)	38 (78%)	30 (70%)	27 (60%)	33 (70%)	31 (72%)	33 (72%)
1	*N* (%)	8 (16%)	11 (26%)	8 (18%)	11 (23%)	10 (23%)	9 (20%)
≥ 2	*N* (%)	3 (6%)	2 (5%)	10 (22%)	3 (6%)	2 (5%)	4 (9%)
Mortality							
Yes	*N* (%)	-	0 (-)	1 (2%)	0 (-)	0 (-)	0 (-)
	Missing	-	-	0	-	-	-
Number of primary care general practice visits							
	Median (IQR)	-	0 [0–1]	0 [0–1]	-	0 [0–1]	0 [0–1]
	Min–max	-	0–13	0–9	-	0–3	0–4
	Missing	-	11	5	-	8	4
0	*N* (%)	-	30 (75%)	29 (67%)	-	34 (83%)	30 (68%)
1	*N* (%)	-	4 (10%)	7 (16%)	-	5 (12%)	4 (9%)
≥ 2	*N* (%)	-	6 (15%)	7 (16%)	-	2 (5%)	10 (23%)
Prescribed medicines							
Any medicine for physical health problems							
Yes	*N* (%)	25 (49%)	23 (56%)	27 (66%)	24 (50%)	20 (53%)	26 (67%)
No	*N* (%)	25 (51%)	18 (44%)	14 (34%)	24 (50%)	18 (47%)	13 (33%)
	Missing	0	10	7	0	11	9
Number of medicines for physical health problems	Mean (SD)	4.6 (4.9)	4.6 (4.0)	3.5 (3.6)	3.1 (2.4)	4.7 (3.5)	3.6 (2.7)
	Median [IQR]	3.0 [2.0–5.0]	3.5 [2.0–7.0]	2.0 [1.0–4.5]	2.0 [1.5–4.5]	4.0 [1.5–7.5]	2.0 [1.0–6.5]
	Min–max	1.0–24.0	1.0–17.0	1.0–17.0	1.0–11.0	1.0–11.0	1.0–9.0
	Missing	27	1	3	24	0	1
Any medicine for mental health problem							
Yes	*N* (%)	24 (47%)	22 (54%)	20 (49%)	21 (44%)	15 (41%)	22 (56%)
No	*N* (%)	27 (53%)	19 (46%)	21 (51%)	27 (56%)	22 (59%)	17 (44%)
	Missing	0	10	7	1	12	9
Number of medicines for mental health problems							
	Mean (SD)	1.5 (0.7)	1.4 (0.7)	1.6 (1.1)	1.4 (0.9)	1.1 (0.4)	1.1 (0.4)
	Median [IQR]	1.0 [1.0–2.0]	1.0 [1.0–2.0]	1 [1–1]	1.0 [1.0–2.0]	1.0 [1.0–1.0]	1 [1–1]
	Min–max	1.0–4.0	1.0–3.0	1.0–5.0	0–4.0	1.0–2.0	1.0–3.0
	Missing	0	3	3	0	0	2
Number of hospitalizations: inpatient							
	Mean (SD)	0.2 (0.7)	0.6 (1.0)	0.8 (1.2)	0.1 (0.2)	0.6 (1.0)	0.5 (0.9)
	Median [IQR]	0 [0–0]	0 [0–1.0]	0 [0–1]	0 [0–0]	0 [0–1.0]	0 [0–1]
	Min–max	0–4.0	0–4.0	0–6	0–1.0	0–5.0	0–4
	Missing	2	4	3	2	2	2
0	*N* (%)	43 (88%)	30 (64%)	27 (60%)	43 (93%)	31 (66%)	33 (72%)
1	*N* (%)	4 (8%)	11 (23%)	8 (18%)	3 (7%)	10 (21%)	9 (20%)
≥ 2	*N* (%)	2 (4%)	6 (13%)	10 (22%)	0 (-)	6 (13%)	4 (9%)
Number of hospitalizations: outpatient							
	Mean (SD)	0.3 (0.8)	0.4 (1.1)	0.3 (0.7)	0.9 (2.4)	0.8 (2.2)	0.8 (2.1)
	Median [IQR]	0 [0–0]	0 [0–0]	0 [0–0]	0 [0–1.0]	0 [0–0]	0 [0–1]
	Min–max	0–4.0	0–5.0	0–4	0–13.0	0–13.0	0–11
	Missing	2	6	3	1	2	2
0	*N* (%)	43 (88%)	38 (84%)	37 (82%)	35 (74%)	36 (77%)	33 (71%)
1	*N* (%)	3 (6%)	2 (4%)	6 (13%)	5 (11%)	4 (9%)	5 (11%)
≥ 2	*N* (%)	3 (6%)	5 (11%)	2 (4%)	7 (15%)	7 (15%)	8 (17%)
COPD Assessment Test^1^							
	Mean (SD)	14.9 (11.7)	14.7 (10.4)	16.2 (10.9)	15.6 (10.4)	16.8 (10.9)	13.8 (9.6)
	Median [IQR]	12.0 [6.0–35.5]	12.0 [7.0–24.0]	14.5 [6.0–27.0]	14.5 [8.0–23.0]	16.0 [7.0–27.0]	13.5 [6.0–22.0]
	Min–max	0–40.0	0–40.0	3.0–40.0	0–40.0	2.0–40.0	0–35.0
	Missing	1	24	10	2	22	16
EQ-5D^2^							
	Mean (SD)	0.463 (0.393)	0.41 (0.43)	0.41 (0.42)	0.394 (0.402)	0.51 (0.36)	0.51 (0.36)
	Missing	0	25	14	3	23	18
EQ-5D Health Thermometer^3^							
	Mean (SD)	45.7 (25.4)	48.1 (19.2)	45.9 (24.8)	38.2 (23.4)	47.0 (24.7)	52.2 (25.6)
	Missing	1	25	12	4	23	18
Fried’s adapted frailty phenotype							
Weight loss: number frail (Frail = lost weight)	*N* (%)	31 (79%)	16 (59%)	29 (76%)	31 (82%)	17 (61%)	25 (76%)
	Missing	12	24	10	10	21	15
Exhaustion: number frail (Frail = felt tired: “More than half the days” and “Nearly every day”)	*N* (%)	39 (76%)	20 (74%)	29 (73%)	41 (87%)	23 (85%)	22 (65%)
	Missing	0	24	8	1	22	14
Physical activity: number frail (Frail = felt tired: “More than half the days” and “Nearly every day”)	*N* (%)	30 (59%)	19 (68%)	23 (56%)	22 (47%)	20 (71%)	20 (61%)
	Missing	0	23	7	1	21	15
Walk time: number frail	*N* (%)	16 (31%)	16 (57%)	12 (31%)	13 (28%)	13 (46%)	12 (36%)
	Missing	0	23	9	1	21	15
Grip strength: number frail	*N* (%)	11 (22%)	11 (39%)	16 (41%)	14 (29%)	11 (41%)	11 (32%)
	Missing	0	23	9	0	22	14
Frailty status							
Non-frail (0 frailty indicators)	*N* (%)	0 (-)	3 (12%)	2 (6%)	0 (-)	0 (-)	2 (6%)
Pre-frail (1–2 frailty indicators)	*N* (%)	18 (46%)	6 (23%)	11 (31%)	16 (42%)	6 (24%)	8 (27%)
Frail (= 3 frailty indicators)	*N* (%)	21 (54%)	17 (65%)	22 (63%)	22 (58%)	19 (76%)	20 (67%)
	Missing	12	25	13	10	24	18
MRC Dyspnoea Scale^4^							
0 = Not troubled by breathless except on strenuous exercise	*N* (%)	17 (33%)	9 (33%)	10 (29%)	14 (29%)	5 (19%)	7 (24%)
1 = Short of breath when hurrying on a level or when walking up a slight hill	*N* (%)	8 (16%)	6 (22%)	11 (32%)	7 (15%)	8 (30%)	7 (24%)
2 = Walks slower than most people on the level, stops after a mile or so, or stops after 15-min walking at own pace	*N* (%)	7 (14%)	6 (22%)	3 (9%)	11 (23%)	4 (15%)	8 (28%)
3 = Stops for breath after walking 100 yards, or after a few minutes on level ground	*N* (%)	10 (20%)	5 (19%)	9 (26%)	7 (15%)	8 (30%)	7 (24%)
4 = Too breathless to leave the house, or breathless when dressing/undressing	*N* (%)	8 (16%)	1 (4%)	1 (3%)	7 (15%)	2 (7%)	0 (-)
	Missing	1	24	14	2	22	19

Data are either mean (SD), median [IQR], or number (%)

For questions with leading question, percentages reflect proportions within the category

*Participants were able to select more than one response

^1^Chronic Obstructive Pulmonary Disease (COPD) Assessment Test (CAT) has a scoring range of 0–40, where higher scores indicate a high severity of COPD

^2^EQ-5D: Quality of life scores range from − 0.59 (worse outcome) to 1.00 (best outcome)

^3^EQ5D Health Thermometer: scores range from 0 (worse outcome) to 100 (best outcome)

^4^MRC Dyspnoea Scale: grades 0 to 4. The higher the grade, the greater the degree of breathlessness related to activity

UC participants saw small decrease in EQ-5D utility scores at 3 (0.41) and 6 (0.41) months compared to the baseline (0.46). Intervention participants saw an increase from baseline value of 0.39 to 0.51 which was sustained at 6 months (Table 2). Similarly, improvement was noticeable in the IG in the visual analog scale. Both UC and IG saw increase in the proportion of participants at 3 and 6 months who were classified as “frail” based on meeting three frailty indicators (Table 2).

Both UC and IG saw an increase in the number of people prescribed medicines for physical and mental health problems at 3 and 6 months compared to baseline (Table 2). Participants in both groups were noted to have increases in prescribed medications for mental health problems (Table 2). However, there was a decrease in the mean number of medicines prescribed per person in 6 months compared to baseline in both UC and IG.

### Social Outcomes

There was a decrease in the proportion of participants sleeping rough in the IG at 3 and 6 months. In the UC, while rough sleeping was decreased at 3 months, it increased at 6 months (Table 3). Similar trends were observed on number of nights slept rough. Involvement with criminal justice (arrests, booked, or charged) in the last 3 months was reduced in the IG group at both follow-up points. In the UC, a reduction was noticed at 3 months but this increased to over baseline values at 6 months. Additional social outcomes data are presented in supplementary material 4.

**Table 3:** Social outcomes

		Usual care			PHOENIx intervention		
		Baseline (*n* = 51)	0–3 months (*n* = 51)	3–6 months (*n* = 48)	Baseline (*n* = 48)	0–3 months (*n* = 49)	3–6 months (*n* = 48)
Type of accommodation							
Hostel (supported)	*N* (%)	24 (47%)	12 (39%)	15 (37%)	21 (44%)	9 (31%)	10 (27%)
Hostel (unsupported)	*N* (%)	8 (16%)	6 (19%)	3 (7%)	9 (19%)	8 (28%)	6 (16%)
No fixable abode	*N* (%)	7 (14%)	2 (6%)	4 (10%)	5 (10%)	2 (7%)	5 (14%)
Other	*N* (%)	7 (14%)	8 (26%)	9 (22%)	3 (6%)	6 (21%)	11 (30%)
Rough sleeping	*N* (%)	2 (4%)	1 (3%)	1 (2%)	7 (15%)	2 (7%)	2 (5%)
Temp furnished flat	*N* (%)	3 (6%)	1 (3%)	2 (5%)	3 (6%)	1 (3%)	3 (8%)
	Missing	0	20	7	0	20	11
In the last 3 months, has participant slept rough in the streets?	Yes	26 (51%)	8 (29%)	16 (42%)	31 (65%)	11 (39%)	11 (46%)
	Missing	0	23	10	0	21	14
If yes, how many nights (approximate if unknown)?	Mean (SD)	23.1 (30.1)	18.9 (32.1)	37.0 (35.3)	29.7 (36.9)	19.8 (25.4)	19.3 (32.4)
	Median [IQR]	10 [2–28]	7 [1–21]	20 [3–77]	7 [3–58]	14 [2–24]	3 [1–24]
	Min–max	1.0–93.0	1–90	1–90	1.0–93.0	1–90	1–90
	Missing	1	1	3	1	0	4
Debt questionnaire							
Does participant currently have any debt/loans?	Yes	17 (33%)	14 (54%)	13 (38%)	7 (15%)	10 (38%)	7 (23%)
	Missing	0	25	14	1	23	18
If yes, how much does participant owe in personal loans, credit cards, or any other loans (£)?	Mean (SD)	2760.8 (5812.4)	1690 (2870)	1800 (2990)	2607.1 (3696.0)	1440 (2410)	1430 (1000)
	Median [IQR]	400.0 [165.0–1575.0]	390 [190–2000]	860 [250–1800]	500.0 [200.0–5000.0]	150 [80–2000]	1250 [1000–2000]
	Min–max	0–20000.0	10–10000	10–10000	50.0–10000.0	30–7000	90–3000
	Missing	5	2	3	0	2	1
In the last 3 months, how much has participant borrowed in loans?	Mean (SD)	23.8 (96.0)	4.5 (15.3)	12.8 (49.7)	16.4 (81.9)	2.7 (12.8)	2.5 (11.2)
	Median [IQR]	0 [0–0]	0 [0–0]	0 [0–0]	0 [0–0]	0 [0–0]	0 [0–0]
	Min–max	0–600	0–70	0–248	0–500	0–60	0–50
	Missing	1	29	25	3	27	29
Criminal justice encounters							
In the last 3 months, has participant been stopped by the police?	Yes	18 (36%)	11 (39%)	14 (35%)	26 (57%)	17 (59%)	16 (47%)
	No	32 (64%)	17 (61%)	26 (65%)	20 (43%)	12 (41%)	18 (53%)
	Missing	1	23	8	2	20	14
If yes, how many times?	Mean (SD)	8.2 (23.5)	2.2 (1.7)	2.9 (2.0)	7.0 (14.4)	6.3 (11.1)	2.7 (4.8)
	Median [IQR]	2 [1–4]	2 [1, 2]	3 [1–5]	2 [2–4]	4 [1–5]	1 [1–1]
	Min–max	1–99	1–6	1–6	1–70	1–45	1–20
	Missing	1	2	3	1	2	1
In the last 3 months, has participant been arrested, booked, or charged for breaking a law?	Yes	8 (16%)	4 (14%)	7 (18%)	14 (31%)	8 (28%)	8 (24%)
	No	42 (84%)	25 (86%)	32 (82%)	31 (69%)	21 (72%)	25 (76%)
	Missing	1	22	9	3	20	15
If yes, how many times?	Mean (SD)	1.3 (0.5)	1.3 (0.5)	1.4 (0.9)	3.1 (5.0)	1.1 (0.4)	1 (-)
	Median [IQR]	1.0 [1.0–2.0]	1.0 [1.0–1.5]	1 [1–1]	1.5 [1.0–2.0]	1.0 [1.0–2.0]	1 [1–1]
	Min–max	1.0–2.0	1–2	1–3	1.0–20.0	1–2	1–1
	Missing	2	0	2	0	1	0
0	*N* (%)	0 (-)	0 (-)	0 (-)	0 (-)	0 (-)	0 (-)
1	*N* (%)	4 (50%)	3 (75%)	4 (80%)	7 (50%)	6 (86%)	8 (100%)
≥ 2	*N* (%)	2 (25%)	1 (5%)	1 (20%)	7 (50%)	1 (14%)	0 (-)
In the last 3 months, has participant been convicted of or pleaded guilty to any charges (other than a minor traffic violation)?	Yes	4 (8%)	4 (13%)	3 (8%)	8 (18%)	5 (17%)	5 (15%)
	No	46 (92%)	26 (87%)	36 (92%)	37 (82%)	24 (83%)	28 (85%)
	Missing	1	21	9	3	20	15
If yes, how many times?	Mean (SD)	1.0 (0)	1.3 (0.6)	1 (-)	2.8 (2.5)	1.0 (-)	1 (-)
	Median [IQR]	1.0 [1.0–1.0]	1 [1, 2]	1 [1–1]	2.0 [1.0–3.5]	1 [1–1]	1 [1–1]
	Min–max	1.0–1.0	1–2	1–1	1.0–8.0	1–1	1–1
	Missing	1	1	1	0	0	0
0	*N* (%)	0 (-)	0 (-)	0 (-)	0 (-)	0 (-)	0 (-)
1	*N* (%)	3 (75%)	2 (67%)	2 (100%)	3 (38%)	5 (100%)	5 (100%)
≥ 2	*N* (%)	0 (-)	1 (33%)	0 (-)	5 (63%)	0 (-)	0 (-)
In the last 3 months has participant ever been under any form of criminal justice supervision, including on probation, in jail, or in prison?	Yes	13 (26%)	5 (17%)	7 (17%)	9 (20%)	5 (17%)	7 (19%)
	No	37 (74%)	25 (83%)	34 (83%)	37 (80%)	25 (83%)	29 (81%)
	Missing	1	21	7	2	19	12
If yes, how many times?	Mean (SD)	1.2 (0.6)	1 (-)	1 (-)	1.0 (0)	1 (-)	1.3 (0.5)
	Median [IQR]	1.0 [1.0–1.0]	1 [1–1]	1 [1–1]	1.0 [1.0–1.0]	1 [1–1]	1 [1, 2]
	Min–max	1.0–3.0	1–1	1–1	1.0–1.0	1–1	1–2
	Missing	3	0	0	2	0	1
0	*N* (%)	0 (-)	0 (-)	0 (-)	0 (-)	0 (-)	0 (-)
1	*N* (%)	9 (69%)	5 (100%)	7 (100%)	7 (78%)	5 (100%)	4 (67%)
≥ 2	*N* (%)	1 (8%)	0 (-)	0 (-)	0 (-)	0 (-)	2 (33%)
Is participant currently on probation?	Yes	8 (16%)	3 (10%)	2 (5%)	10 (21%)	1 (3%)	1 (3%)
	No	43 (84%)	26 (90%)	36 (95%)	34 (77%)	28 (97%)	33 (97%)
	Missing	0	22	10	4	20	14
If yes, for how long (days)?	Mean (SD)	169 (147)	370 (511)	198 (237)	227 (205)	12 (-)	365 (-)
	Median [IQR]	124 [42–365]	369 [8–730]	198 [114–281]	165 [90–365]	12 [12–12]	365 [365–365]
	Min–max	3.0–365.0	8–730	30–365	20.0–558.0	12–12	365–365
	Missing	1	1	0	4	0	0
How many days in the last 3 months has participant spent in jail or prison in total?	Mean (SD)	43.9 (33.9)	0 (-)	10	6.8 (15.2)	6.8 (20.0)	10
	Median [IQR]	47.5 [14.0–70.0]	0 [0–0]	9.1 (28.8)	0 [0–0]	0 [0–0]	17.0 (32.1)
	Min–max	0–88.0	0–0	0 [0–0]	0–34.0	0–60	0 [0–21]
	Missing	43	45	0–91	43	40	0–92
Since age 18, has participant ever been in jail or in prison?	Yes	37 (73%)	-	-	38 (79%)	-	-
	No	14 (27%)	-	-	8 (17%)	-	-
	Missing	0	-	-	2	-	-
If yes, how many times?	Mean (SD)	7.4 (23.9)	-	-	16.5 (20.2)	-	-
	Median [IQR]	7.0 [2.0–22.0]	-	-	10.0 [5.0–20.0]	-	-
	Min–max	1.0–100.0	-	-	1.0–100.0	-	-
	Missing	1	-	-	1	-	-

Data are either mean (SD), median [IQR], or number (%)

For questions with leading question, percentages reflect proportions within the category

### Addiction-Specific Outcomes

For problem drug use, 77% and 74% of participants in the UC and IG had OST medicines prescribed at baseline (Table 4). This decreased at 6-month follow-up to 71% and 61% respectively in the two groups. When specifically looking into methadone prescribing, both UC and IG group saw a reduction in the number of people prescribed methadone at both follow-up points (Supplementary material 5). The proportion of participants currently using heroin decreased across both groups in the follow-up period. There were eight participants (four control and four intervention group) who reported current heroin use at baseline and not using heroine at the 6-month follow-up. These changes, however, were not linked to new OST prescribing (Supplementary material 5). The proportion of participants consuming alcohol on a daily basis increased at both follow-up points. Small reductions in smoking rates were observed at 6 months across both groups (Supplementary material 5).

**Table 4:** Addiction-specific outcomes

		Usual care			PHOENIx intervention		
		Baseline (*n* = 51)	0–3 months (*n* = 51)	3–6 months (*n* = 48)	Baseline (*n* = 48)	0–3 months (*n* = 49)	3–6 months (*n* = 48)
Number experiencing drug overdoses not requiring emergency department visit							
	Mean (SD)	-	-	0.4 (0.5)	-	-	0.3 (0.5)
	Median (IQR)	-	-	0 [0–1]	-	-	0 [0–0.5]
	Min–Max	-	0	0–1	-	0	0–1
	Missing	-	47	46	-	47	45
0	*N* (%)	-	4 (100%)	3 (60%)	-	2 (100%)	3 (75%)
1	*N* (%)	-	0 (-)	2 (40%)	-	0 (-)	1 (25%)
≥ 2	*N* (%)	-	0 (-)	0 (-)	-	0 (-)	0 (-)
Number of ODs in the past 3 months							
	Mean (SD)	0.1 (0.5)	0.3 (0.8)	0.3 (1.1)	0.3 (0.8)	0.1 (0.4)	0.1 (0.3)
	Median [IQR]	0 [0–0]	0 [0–0]	0 [0–0]	0 [0–0]	0 [0–0]	0 [0–0]
	Min–max	0–2.0	0–4	0–7	0–4.0	0–2	0–1
	Missing	1	23	6	1	21	14
0	*N* (%)	46 (92%)	23 (82%)	36 (86%)	36 (77%)	26 (93%)	30 (88%)
1	*N* (%)	1 (2%)	4 (14%)	4 (10%)	9 (19%)	1 (4%)	4 (12%)
≥ 2	*N* (%)	3 (6%)	1 (4%)	2 (5%)	2 (4%)	1 (4%)	0 (-)
From pharmacy records, has participant collected 80% of daily dose in the last 3 months?	Yes	19 (86%)	7 (88%)	7 (64%)	20 (83%)	2 (29%)	3 (43%)
	No	3 (14%)	1 (13%)	4 (36%)	4 (17%)	5 (71%)	4 (57%)
	Missing	29	43	37	24	42	41
Drug use							
Heroin: current	Yes	16 (31%)	8 (29%)	12 (30%)	21 (44%)	8 (30%)	11 (34%)
	No	35 (69%)	20 (71%)	28 (70%)	27 (56%)	19 (70%)	21 (66%)
	Missing	0	23	8	0	22	16
Age first used	Mean (SD)	24.8 (6.7)	-	-	21.8 (5.2)	-	-
	Median [IQR]	26.0 [18.0–31.0]	-	-	20.5 [18.0–25.5]	-	-
	Min–max	14.0–35.0	-	-	15.0–33.0	-	-
	Missing	1	-	-	1	-	-
Cocaine: current	Yes	24 (47%)	14 (48%)	18 (46%)	26 (54%)	12 (44%)	16 (50%)
	No	27 (53%)	15 (52%)	21 (54%)	22 (46%)	15 (56%)	16 (50%)
	Missing	0	22	9	0	22	16
Age first used	Mean (SD)	25.5 (8.3)	-	-	23.1 (8.6)	-	-
	Median [IQR]	25.5 [18.0–32.0]	-	-	20.5 [18.0–25.0]	-	-
	Min–max	10.0–40.0	-	-	14.0–45.0	-	-
	Missing	2	-	-	0	-	-
Street Valium: current	Yes	10 (20%)	8 (29%)	12 (31%)	4 (8%)	8 (31%)	7 (23%)
	No	40 (80%)	20 (71%)	27 (69%)	44 (92%)	18 (69%)	24 (77%)
	Missing	1	23	9	0	23	17
Age first used	Mean (SD)	28.7 (12.7)	-	-	18.0 (3.6)	-	-
	Median [IQR]	27.0 [16.0–40.0]	-	-	19.0 [14.0–21.0]	-	-
	Min–max	15.0–44.0	-	-	14.0–21.0	-	-
	Missing	1	-	-	1	-	-
Spice: current	Yes	3 (6%)	4 (14%)	4 (10%)	5 (10%)	2 (8%)	3 (10%)
	No	46 (94%)	24 (86%)	35 (90%)	43 (90%)	24 (92%)	28 (90%)
	Missing	2	23	9	0	23	17
Age first used	Mean (SD)	40.7 (6.0)	-	-	35.0 (13.1)	-	-
	Median [IQR]	40.0 [35.0–47.0]	-	-	33.5 [25.5–52.0]	-	-
	Min–max	35.0–47.0	-	-	21.0–52.0	-	-
	Missing	0	-	-	1	-	-
Street gabapentoids: current	Yes	6 (12%)	2 (7%)	4 (11%)	4 (8%)	4 (15%)	2 (6%)
	No	44 (88%)	26 (93%)	34 (89%)	44 (92%)	22 (85%)	29 (94%)
	Missing	1	23	10	0	23	17
Age first used	Mean (SD)	42.8 (9.3)	-	-	29.0 (3.6)	-	-
	Median [IQR]	40.0 [40.0–47.0]	-	-	30.0 [25.0–32.0]	-	-
	Min–max	31.0–56.0	-	-	25.0–32.0	-	-
	Missing	1	-	-	1	-	-
Cannabis: current	Yes	11 (22%)	8 (29%)	12 (31%)	20 (42%)	12 (48%)	8 (26%)
	No	40 (78%)	20 (71%)	27 (69%)	28 (48%)	13 (52%)	23 (74%)
	Missing	0	23	9	0	24	17
Age first used	Mean (SD)	17.2 (10.5)	-	-	16.8 (8.7)	-	-
	Median [IQR]	13.5 [12.0–15.0]	-	-	14.0 [12.0–16.0]	-	-
	Min–max	8.0–38.0	-	-	11.0–48.0	-	-
	Missing	1	-	-	1	-	-
Problem alcohol use							
Does participant drink daily	Yes	8 (16%)	5 (18%)	9 (23%)	10 (21%)	9 (32%)	9 (29%)
	No	42 (84%)	23 (45%)	30 (77%)	37 (79%)	19 (68%)	22 (71%)
	Missing	1	23	9	1	21	17
Ever had a detox/hospital stay/rehab for alcohol?	Yes	10 (20%)	1 (4%)	2 (5%)	9 (19%)	1 (4%)	1 (3%)
	No	39 (80%)	27 (96%)	36 (95%)	38 (81%)	26 (96%)	29 (97%)
	Missing	2	23	10	1	22	18
Previous seizures or hallucinations related to alcohol withdrawal?	Yes	12 (24%)	1 (4%)	1 (3%)	14 (30%)	5 (19%)	3 (10%)
	No	38 (76%)	27 (96%)	36 (97%)	33 (70%)	22 (81%)	27 (90%)
	Missing	1	23	11	1	22	18
Units/week	Mean (SD)	30.0 (94.9)	16.4 (34.3)	43.7 (112.6)	38.7 (84.0)	63.1 (112.2)	54.6 (96.2)
	Median [IQR]	0.0 [0.0–0.5]	0 [0–16]	0 [0–6]	0.0 [0.0–20.0]	0 [0–96]	0 [0–101.5]
	Min–max	0–560.0	0–136	0–560	0–315.0	0–350	0–350
	Missing	7	24	17	6	23	21
Smoking							
Current tobacco smoker?	Yes	45 (88%)	26 (90%)	33 (85%)	44 (94%)	27 (96%)	30 (91%)
	No	4 (8%)	2 (7%)	3 (8%)	2 (4%)	1 (4%)	2 (6%)
	Ex-smoker	2 (4%)	1 (3%)	3 (8%)	1 (2%)	0 (-)	1 (3%)
	Missing	0	22	9	1	21	15
If current or ex-smoker, age started	Mean (SD)	13.0 (4.0)	-	-	14.6 (6.9)	-	-
	Median [IQR]	14.0 [11.0–15.0]	-	-	14.0 [11.5–15.5]	-	-
	Min–max	0–21.0	-	-	6.0–48.0	-	-
	Missing	18	-	-	13	-	-
If yes, no. of cigarettes a day	Mean (SD)	11.0 (16.2)	12.5 (20.3)	12.1 (11.2)	10.1 (11.6)	11.9 (13.9)	9.3 (13.6)
	Median [IQR]	8.0 [0–15.0]	8 [0–20]	9 [1–20]	5.0 [0–20.0]	10 [1–15]	6 [0–15]
	Min–max	0–100.0	0–100	0–40	0–40.0	0–60	0–60
	Missing	2	2	1	2	1	3
If yes, no. of roll ups a day	Mean (SD)	7.2 (14.0)	3.5 (6.8)	6.7 (14.5)	5.7 (9.2)	3.5 (7.6)	7.1 (11.3)
	Median [IQR]	0 [0–10.0]	0 [0–10]	0 [0–10]	0 [0–10.0]	0 [0–3]	0 [0–11]
	Min–max	0–80.0	0–25	0–70	0–30.0	0–30	0–39
	Missing	4	2	6	2	1	8
Does participant vape (e-cigarettes)?	Yes	19 (39%)	12 (43%)	19 (49%)	13 (28%)	13 (50%)	10 (33%)
	No	30 (61%)	16 (57%)	20 (51%)	34 (72%)	13 (50%)	20 (66%)
	Missing	2	23	9	1	23	18
Currently on NRT?	Yes	4 (8%)	1 (4%)	4 (11%)	5 (11%)	1 (4%)	0 (-)
	No	44 (92%)	24 (96%)	32 (89%)	41 (89%)	24 (96%)	27 (100%)
	Missing	3	26	12	2	24	21
Ambulance data							
Participant used an ambulance in past 3 months?							
	Yes	-	12 (31%)	14 (33%)	-	10 (24%)	7 (18%)
	No	-	27 (69%)	28 (67%)	-	31 (76%)	33 (83%)
	Missing	-	12	6	-	8	8
If yes, how many times?	Mean (SD)	-	1.8 (1.3)	1.9 (1.4)	-	1.3 (0.7)	1.4 (0.8)
	Median [IQR]	-	1 [1–2.5]	1 [1, 2]	-	1 [1–1]	1 [1, 2]
	Min–max	-	1–5	1–5	-	1–3	0–3
	Missing	-	0	0	-	0	0
0	*N* (%)	-	0 (-)	0 (-)	-	0 (-)	0 (-)
1	*N* (%)	-	8 (67%)	9 (64%)	-	8 (80%)	5 (71%)
≥ 2	*N* (%)	-	4 (33%)	5 (36%)	-	2 (20%)	2 (29%)

PHOENIx team made a number of referrals to different services including mental health services, alcohol and drug recovery services, and GPs (Supplementary material 5). Based on the data available from all 25 IG from one study center (Glasgow), pharmacists directly prescribed 118 medicines, discussed physical health with other professionals 154 times, and discussed participant’s mental health 281 times. In Glasgow, as pharmacists were not permitted to prescribe OST or other treatments for substance use, instead of initiating or continuing OST on the same day, they discussed or made appointments for participants 92 times, or asked participants to make the journey local alcohol and drug recovery services 29 times.

## Discussion

Integrated approaches to health, housing, and social support for PEH is emphasized by health policies including recent NICE Guideline on care for PEH [21]. However, limited attempts have been made to develop and evaluate integrated approaches to care using a gold standard methodology. To our knowledge, this is the first community pharmacy-based multicenter randomized controlled trial that achieves this. The study was able to exceed progression criteria for a definitive trial in terms of recruitment, retention, intervention adherence, and collection of outcome data [26].

Our original rationale for conducting this RCT in community pharmacy—that it would be a positive place to engage patients—was supported by the success in participant recruitment. Pharmacy staff used their acquaintance, prescription records, and informal conversations based on their rapport to identify suitable persons to invite. We also used prescription records to identify people who were unable to collect prescribed medicines in person in pharmacy due to health problems, and approached them via the third sector worker ensuring equalities in opportunities for participation in the trial.

The number of participating community pharmacies was increased and the recruitment period was extended in one site to ensure recruitment targets were achieved. Prior experience of researchers and the PHOENIx delivery team in one site, due to experiences with a previous RCT, is likely to have led to a more rapid recruitment rate and higher numbers of contacts with participants in the PHOENIx arm. It may be prudent in a subsequent definitive trial, to schedule a run-in period where researchers and intervention staff become familiar with local venues and the local NHS organizational context.

Researchers, and intervention staff, were recruited to the study team on the strength of their demonstrable empathy, street sense, active listening, non-judgemental attitude, and relationship building skills. HVCSEs provide unconditional support to PEH and this ethos was also important for all staff (researchers and intervention team) involved in delivering PHOENIx. We suggest these skills are important factors in the success of recruitment and follow-up, given the past and ongoing traumatic life experiences of PEH that impact on emotional regulation, executive functioning, and forming secure relationships [10].

Three-month follow-up was impacted negatively due to the temporary absence of the researcher and third sector staff in one site. In addition, some delays in patient recruitment in one site led to recruitment and 3-month follow-up of some participants ongoing at the same time which added additional pressure to the field researchers. Assistance from the wider network of third sector organizations and agencies were received to enable 6-month follow-up which maximized response rate. When participants had moved pharmacies, temporary accommodation, moved to a different city, or were imprisoned, this brought about anticipated governance and data linkage barriers. However, healthcare utilization data was successfully obtained with a high success rate in both sites. In Scotland, these were facilitated through Community Health Index (CHI) search records from the NHS databases. In England, we approached individual GP practices, hospitals, and emergency departments—albeit this was labor intensive.

The intervention showed potential in preventing deterioration of emergency health needs as excess ED presentations were reduced in the intervention group. Both the utility scores and visual analog scale (VAS) showed potential improvement in quality of life. No remarkable trends were observed across secondary outcomes such as routine hospital admissions and frailty (meeting three frailty indicators). Signals of improvement across social outcomes such as rough sleeping and involvement with criminal justice system were encouraging. In particular, the third sector worker offered express referral to housing, furnishing, and tenancy maintenance support where needed to prevent rough sleeping.

The above findings corroborate with the evaluation of PHOENIx model of care in other settings and a pilot RCT of PHOENIx model of care for PEH with a history of overdose [17, 31]. Sparse literature using RCT evaluation of care models prohibits wider comparison of results.

The range of consultation frequencies across participants in both settings is noteworthy. Having a responsive intervention team with dedicated time and connections to meet participants’ fluctuating needs and engagement styles was seen as important. Widening the scope of practice for clinicians on outreach may offer even greater immediate help, if mobile teams are linked to General Practices [37]. Whether the PHOENIx model of care, provided by highly qualified independent prescribing pharmacists and HVCSE worker, is capable of being rolled out at scale and pace to the UK’s homeless population is unclear, due to workforce challenges and costs. A health economic analysis and definitive trial will help clarify whether there is merit in expansion of PHOENIx at scale, or whether alternative models, e.g., HVCSE with a GP linked Health Care Assistant conducting regular outreach to support diagnosis and treatment, might be a generalizable approach remains to be explored.

### Strengths and Limitations

Community pharmacies have been referred to as “untapped” resource in terms of their potential to offer opportunistic intervention including advice, referral, and urgent treatment to mitigate health inequalities [38]. Examples of emerging roles include screening and treatment of blood-borne viruses including hepatitis C [39] and HIV [40] in marginalized communities. The results of this study provide positive signs that community pharmacy can be a suitable avenue to identify and offer support to people facing the worst health outcomes in society. The intervention was delivered predominantly in the participants’ choice of venue out with the community pharmacy, underscoring the importance of having a nimble team, ready to go to the participant. This, together with strong links to the local (specialist inclusion health) GP practices, the strength of the relationship between PHOENIx and participants, and the generalist skills of the pharmacist for managing multimorbidities, is likely to be an important feature to retain in an intervention in a definitive trial.

Given the pilot nature of the study, it was not set up to undertake hypothesis testing so signs of improved clinical outcomes need further exploration in a full RCT. There were missing data across different data fields owing to non-response in the face-to-face/telephone follow-up and data requests from other service providers. Some of the case record forms completed also had missing data fields as often the researchers could not complete the extensive data collection form in one session with the participant. Some clinical measures were missing as we had to utilize community researchers (third sector workers) to undertake clinical observations (after additional training) such as blood pressure and BMI checks. These were not always completed. Both settings were urban, and given the rise in numbers of PEH in remote and rural areas, and variations in presentations in coastal areas, there may be merit in including PEH from rural and coastal areas in the subsequent RCT.

Variations in the nature and extent of support given to participants between settings, more work is needed to understand the reasons, e.g., pharmacist training and experience. Our qualitative evaluation (under review at the time of the manuscript preparation) may shed light on these variations, from the perspective of participants and stakeholders and PHOENIx delivery teams. Glasgow is a smaller city than Birmingham with fewer places for researchers and the intervention staff to seek and follow-up participants. Along with prior experience of delivery of the PHOENIx feasibility study in Glasgow and an established local support network, it has likely led to higher follow-up rates in Glasgow compared to Birmingham. A “run in” period, as occurs in clinical trials, may be indicated, to ensure delivery teams are able to navigate local complex systems, prescribing protocols, housing referrals, and familiarize themselves with local HVCSE venues. A future trial will investigate the roles of geographical/center-level practice variations in any observed differences across outcomes.

VCSE workers’ rapport and relationship with participants was key to successful engagement and delivery of interventions in both settings. Some participants were imprisoned during the follow-up period and it was only possible to follow-up some of these participants in prison. The intervention team and the researchers also had difficulties in engaging with participants who moved out of the local geographical area. We realized that researchers found it more challenging to follow-up UG participants compared to IG due to the lack of intervention team engagement with the former group. Our intervention team realized that there could be multiple and often overlapping professionals attempting to assist PEH but functioning separately. Strengthening collaboration within local care services is key. Additional funding within the trial to support social wellbeing such as gym memberships, skills training, clothing, furniture, and subsistence could promote participant engagements.

All study researchers and intervention staff had street sense, were trauma-informed, and applied harm reduction principles. Community pharmacies who participated in the study also had acquaintance with the study population as they were providers of OST and needle exchange services, and were located in urban centers/nearby where PEH reside.

The intervention team aimed for weekly contacts for each participant which would equate to six appointments a day. However, intervention staff often had to spend half or the whole day with a participant who had high needs, for example, needed help with bank account opening or accompanying to a further healthcare appointment. Such longer contacts were key to promote engagement and develop rapport between the intervention team and the participants. In one of the study sites, there was a temporary absence of support worker which may have led to some participants disengaging. Future studies should ideally be better resourced to cover staff sickness.

### Recommendations for Future Definitive Trial

While recruitment was primarily opportunistic based on who was presenting to the pharmacies on the recruitment days, a future trial could also consider identifying PEH who are not physically able to walk to the pharmacy to receive services.

PHOENIx teams supported some participants intensively, and others less intensively. Future studies can benefit from early identification and prioritization for provision of additional support including assertive outreach for PEH with high needs but least likely to engage with the intervention. Amendments in local governance including prescribing rights (e.g., for substance use disorder treatment) and better skill mix within intervention team (e.g., availability of prescribers on outreach with diverse clinical competencies) could minimize barriers to rapid provision of treatment and adaptation of intervention to PEH needs.

Given the limited space and availability of consultation rooms, some of the baseline and follow-up assessments by the researchers and intervention staff visits were conducted in community venues in close proximity to the pharmacy where both the researcher and the participant felt comfortable. Our experience also suggests that recruitment in wintertime can also be challenging due to low footfall in pharmacies.

Lessons were learned with regard to challenges in following up participants who were in prison, or moved out of the local geographical area. In Scotland, prison personnel helped the study team by accommodating visits by the study team for follow-up. However, in England, governance barriers did not allow visits to take place during the study period. Utilizing Rough Sleepers Outreach Team from local authorities to find participants would be key given their wider approach, access to participant accommodation data or “know how” of where a participant was known to bed down or beg. Pharmacy records were less useful in finding participants when they had moved to a different pharmacy as details of the new provider were not available. A future study should also gather information on participant whereabouts (including UC) more frequently during follow-up rather than at 3 and 6 months, and follow-up for longer, given the likely median duration of homelessness of over 20 years in some cohorts [17].

Future studies could also utilize the community pharmacy team to directly offer clinical assessment and prescribing for PEH as their roles in this study was limited to participant identification, referral, and provision of usual care. There is a potential to upskill pharmacy staff in research capacity such as in obtaining informed consent to facilitate recruitment of participants out of hours and to maximize efficiency in larger trials. This would require advanced agreement with clinical and information governance in local NHS areas, given that community pharmacists in the UK are contractors rather than NHS employees (PHOENIx pharmacists). PHOENIx teams also had support from Pharmacy leads in NHS Primary Care (Glasgow) and Acute (Birmingham) which supported information and clinical governance, both of which are pre-requisites to safe and effective clinical practice, and lacking in the organizational structure of community pharmacy in Glasgow and Birmingham. With prescribing rights being expanded to pharmacists in the UK [41], their roles in provision of generalist care for vulnerable populations have the potential to mitigate health inequalities.

## Conclusion

This community pharmacy-based PHOENIx multicenter RCT was able to recruit, follow-up, and collect healthcare utilization data from an unselected cohort of PEH across two urban settings exceeded the progression criteria. PHOENIx community pharmacy RCT merits progression into a definitive trial which will evaluate the effectiveness and cost-effectiveness of the PHOENIx community pharmacy intervention.

## Supplementary Information

Below is the link to the electronic supplementary material.

Supplementary file1 (DOCX 91 KB)

## Data Availability

Only scientifically sound proposals from appropriately qualified Research Groups will be considered for data sharing. The request will be reviewed by the BCTU Data Sharing Committee in discussion with the Chief Investigators.
