# Supplementary material for: Pharmacy Homeless Outreach Engagement Non-medical Independent Prescribing Rx (PHOENIx) Community Pharmacy-Based Pilot Randomized Controlled Trial
Source: J Urban Health. 2025 Jun 9;102(3):540–63. doi: 10.1007/s11524-025-00981-0 (PMC12279658; doi:10.1007/s11524-025-00981-0)
Supplement: Supplementary file 1 — Supplementary file1 (DOCX 91 KB) [file 11524_2025_981_MOESM1_ESM.docx]

**Supplementary material 1: Recruitment number in each pharmacy**

| **Site** | **Usual Care** | **PHOENIx Intervention** | **TOTAL** |
| --- | --- | --- | --- |
| Pharmacy 1 (Glasgow) | 17 | 18 | **35** |
| Pharmacy 2 (Glasgow) | 8 | 7 | **15** |
| Pharmacy 1 (Birmingham) | 19 | 19 | **38** |
| Pharmacy 2 (Birmingham) | 5 | 1 | **6** |
| Pharmacy 3 (Birmingham) | 2 | 4 | **6** |
| **TOTAL** | **51** | **49** | **100** |

**Supplementary material 2: Additional data- Clinical Outcomes**

|  |  | **Usual Care** | | | **PHOENIx Intervention** | | |
| --- | --- | --- | --- | --- | --- | --- | --- |
|  |  | **Baseline**  **(n=51)** | **0-3 Months**  **(n=51)** | **3-6 Months**  **(n=48)** | **Baseline**  **(n=48)** | **0-3 Months**  **(n=49)** | **3-6 Months**  **(n=48)** |
| Cause of Emergency Department visits (Top 5 highest frequency reported)* |  |  |  |  |  |  |  |
| Overdose | N (%) | 2 (4%) | 4 (33%) | 0 (-) | 3 (6%) | 2 (17%) | 0 (-) |
| Assaulted | N (%) | 2 (4%) | 0 (-) | 5 (28%) | 1 (2%) | 0 (-) | 1 (8%) |
| Self-referral | N (%) | 1 (2%) | 0 (-) | 0 (-) | 2 (4%) | 0 (-) | 0 (-) |
| Intoxicated | N (%) | 3 (6%) | 0 (-) | 0 (-) | 0 (-) | 0 (-) | 0 (-) |
| Mental Health | N (%) | 1 (2%) | 0 (-) | 0 (-) | 1 (2%) | 0 (-) | 0 (-) |
| Seizure | N (%) | 0 (-) | 3 (25%) | 7 (39%) | 0 (-) | 1 (8%) | 1 (8%) |
| Fall | N (%) | 0 (-) | 1 (8%) | 0 (-) | 0 (-) | 2 (17%) | 0 (-) |
| DVT | N (%) | 0 (-) | 0 (-) | 0 (-) | 0 (-) | 3 (25%) | 0 (-) |
| Abscess | N (%) | 0 (-) | 1 (8%) | 0 (-) | 0 (-) | 1 (8%) | 0 (-) |
| Foot injury | N (%) | 0 (-) | 0 (-) | 3 (17%) | 0 (-) | 0 (-) | 2 (15%) |
| Groin pain | N (%) | 0 (-) | 0 (-) | 3 (17%) | 0 (-) | 0 (-) | 0 (-) |
| Collapsed | N (%) | 0 (-) | 0 (-) | 1 (6%) | 0 (-) | 0 (-) | 0 (-) |
|  | Missing | 0 | 3 | 1 | 0 | 3 | 8 |
| Cause of Primary Care General Practice visits (Top 5 highest frequency reported)* |  |  |  |  |  |  |  |
|  | DVT | - | 0 (-) | 0 (-) | - | 3 (43%) | 0 (-) |
|  | Leg Pain | - | 2 (20%) | 2 (14%) | - | 1 (14%) | 2 (14%) |
|  | Stroke clinic | - | 1 (10%) | 1 (7%) | - | 0 (-) | 0 (-) |
|  | Low mood | - | 1 (10%) | 0 (-) | - | 0 (-) | 0 (-) |
|  | Itching all over | - | 0 (-) | 0 (-) | - | 1 (14%) | 0 (-) |
|  | COPD | - | 0 (-) | 0 (-) | - | 0 (-) | 2 (14%) |
|  | Abscess | - | 0 (-) | 0 (-) | - | 0 (-) | 2 (14%) |
|  | Numb hand | - | 0 (-) | 2 (14%) | - | 0 (-) | 0 (-) |
|  | Missing | - | 6 | 9 | - | 2 | 8 |
| Medicines for physical health problems* |  |  |  |  |  |  |  |
| Analgesic | N (%) | 9 (18%) | 8 (35%) | 10 (37%) | 7 (15%) | 11 (55%) | 14 (54%) |
| Respiratory | N (%) | 7 (14%) | 8 (35%) | 7 (26%) | 8 (17%) | 9 (45%) | 12 (46%) |
| Nutrition and anaemia | N (%) | 8 (16%) | 9 (39%) | 11 (41%) | 4 (8%) | 11 (55%) | 10 (38%) |
| Upper gastrointestinal | N (%) | 9 (18%) | 5 (22%) | 11 (41%) | 3 (6%) | 4 (20%) | 8 (31%) |
| Antiepileptic | N (%) | 5 (10%) | 3 (13%) | 4 (15%) | 4 (8%) | 4 (20%) | 5 (19%) |
| Antibacterial/antifungal | N (%) | 4 (8%) | 7 (30%) | 6 (22%) | 4 (8%) | 5 (25%) | 4 (15%) |
| Topical for skin condition | N (%) | 4 (8%) | 3 (13%) | 3 (11%) | 4 (8%) | 6 (30%) | 6 (23%) |
| Antihypertensive | N (%) | 5 (10%) | 1 (4%) | 3 (11%) | 2 (4%) | 1 (5%) | 2 (8%) |
| Antiplatelet | N (%) | 3 (6%) | 4 (17%) | 3 (11%) | 2 (4%) | 2 (10%) | 4 (15%) |
| Laxative | N (%) | 2 (4%) | 2 (9%) | 2 (7%) | 1 (2%) | 0 (-) | 0 (-) |
| Statin | N (%) | 3 (6%) | 3 (13%) | 3 (11%) | 0 (-) | 0 (-) | 0 (-) |
| Antiretroviral | N (%) | 1 (2%) | 2 (9%) | 1 (4%) | 1 (2%) | 2 (10%) | 0 (-) |
| Diabetes | N (%) | 2 (4%) | 1 (4%) | 1 (4%) | 0 (-) | 0 (-) | 0 (-) |
| Diuretic | N (%) | 0 (-) | 0 (-) | 0 (-) | 1 (2%) | 0 (-) | 1 (4%) |
| Hormone replacement therapy | N (%) | 1 (2%) | 1 (4%) | 0 (-) | 0 (-) | 0 (-) | 0 (-) |
| Drug for movement disorder | N (%) | 0 (-) | 0 (-) | 0 (-) | 1 (2%) | 0 (-) | 0 (-) |
| Nocturnal Leg Cramps | N (%) | 0 (-) | 0 (-) | 0 (-) | 0 (-) | 0 (-) | 0 (-) |
| Sex hormone | N (%) | 0 (-) | 0 (-) | 0 (-) | 0 (-) | 0 (-) | 0 (-) |
| Medicines for mental health problems* |  |  |  |  |  |  |  |
| Antidepressant | N (%) | 17 (71%) | 7 (32%) | 10 (50%) | 14 (67%) | 5 (33%) | 4 (18%) |
| Antipsychotic | N (%) | 7 (29%) | 13 (59%) | 15 (75%) | 7 (33%) | 11 (79%) | 17 (77%) |
| Anxiolytic | N (%) | 4 (17%) | 1 (5%) | 4 (20%) | 1 (5%) | 0 (-) | 1 (5%) |
| Cause of inpatient hospitalisations (Top 5 highest frequency reported)* |  |  |  |  |  |  |  |
| Intoxicated | N (%) | 4 (8%) | 0 (-) | 0 (-) | 0 (-) | 0 (-) | 0 (-) |
| Sepsis | N (%) | 2 (4%) | 0 (-) | 0 (-) | 0 (-) | 0 (-) | 0 (-) |
| Gave birth | N (%) | 1 (2%) | 0 (-) | 0 (-) | 0 (-) | 0 (-) | 0 (-) |
| Infectious disease | N (%) | 1 (2%) | 0 (-) | 0 (-) | 0 (-) | 0 (-) | 0 (-) |
| Respiratory | N (%) | 1 (2%) | 0 (-) | 0 (-) | 0 (-) | 0 (-) | 0 (-) |
| DVT | N (%) | 0 (-) | 1 (2%) | 0 (-) | 0 (-) | 2 (4%) | 0 (-) |
| Abscess | N (%) | 0 (-) | 1 (2%) | 2 (4%) | 0 (-) | 1 (2%) | 0 (-) |
| Cellulitis | N (%) | 0 (-) | 1 (2%) | 0 (-) | 0 (-) | 1 (2%) | 0 (-) |
| Biopsies | N (%) | 0 (-) | 0 (-) | 0 (-) | 0 (-) | 1 (2%) | 0 (-) |
| Laceration to forehead | N (%) | 0 (-) | 1 (2%) | 0 (-) | 0 (-) | 1 (2%) | 0 (-) |
| Seizure | N (%) | 0 (-) | 0 (-) | 5 (11%) | 0 (-) | 0 (-) | 1 (2%) |
| Alcohol withdrawal | N (%) | 0 (-) | 0 (-) | 0 (-) | 0 (-) | 0 (-) | 2 (4%) |
| Foot Injury | N (%) | 0 (-) | 0 (-) | 2 (4%) | 0 (-) | 0 (-) | 0 (-) |
| Dyspnoea | N (%) | 0 (-) | 0 (-) | 1 (2%) | 0 (-) | 0 (-) | 0 (-) |
| Cause of outpatient hospitalisations (Top 5 7 (15%)highest frequency reported)* |  |  |  |  |  |  |  |
| Infectious Disease |  | 3 (6%) | 4 (9%) | 0 (-) | 9 (19%) | 0 (-) | 4 (9%) |
| Gastroenterology |  | 0 (-) | 0 (-) | 0 (-) | 3 (6%) | 1 (2%) | 2 (4%) |
| Chiropody |  | 1 (2%) | 0 (-) | 0 (-) | 1 (2%) | 0 (-) | 4 (9%) |
| Ophthalmology |  | 1 (2%) | 0 (-) | 0 (-) | 1 (2%) | 0 (-) | 0 (-) |
| Orthopaedics |  | 2 (4%) | 0 (-) | 0 (-) | 0 (-) | 0 (-) | 0 (-) |
| Podiatry |  | 0 (-) | 3 (7%) | 0 (-) | 0 (-) | 4 (9%) | 0 (-) |
| Plastic Surgery |  | 0 (-) | 0 (-) | 0 (-) | 0 (-) | 2 (4%) | 0 (-) |
| Community Stroke Team |  | 0 (-) | 2 (4%) | 0 (-) | 0 (-) | 0 (-) | 0 (-) |
| X-ray/Scan |  | 0 (-) | 0 (-) | 1 (2%) | 0 (-) | 0 (-) | 4 (9%) |
| Plastic Surgery |  | 0 (-) | 0 (-) | 0 (-) | 0 (-) | 0 (-) | 1 (2%) |
| **Blood pressure: Systolic (mmHg)** |  |  |  |  |  |  |  |
|  | Mean (SD) | 125.1 (18.4) | 122.7 (20.6) | 124.9 (25.3) | 118.7 (16.1) | 120.1 (16.7) | 116.6 (15.9) |
|  | Median [IQR] | 124.0  [113.0 - 133.0] | 119.5  [105.5 - 132.5] | 121.0  [111.5 – 136.0] | 116.5  [108.0 - 130.0] | 118.0  [107.0 - 131.0] | 117.5  [105.0 – 128.0] |
|  | Min - Max | 92.0 – 194.0 | 93.0 – 171.0 | 67.0 – 186.0 | 71.0 – 149.0 | 95.0 – 153.0 | 80.0 – 154.0 |
|  | Missing | 5 | 27 | 19 | 6 | 24 | 23 |
| **Blood pressure: Diastolic (mmHg)** |  |  |  |  |  |  |  |
|  | Mean (SD) | 79.1 (11.9) | 77.0 (11.3) | 79.7 (12.3) | 77.6 (14.2) | 78.8 (11.2) | 81.2 (15.6) |
|  | Median [IQR] | 79.0  [71.0 – 85.0] | 75.0  [70.0 - 86.0] | 80.0  [72.0 – 87.5] | 77.0  [73.0 – 84.0] | 77.0  [89.0 - 70.0] | 80.0  [72.0 – 85.0] |
|  | Min - Max | 53.0 – 118.0 | 56.0 – 98.0 | 58.0 – 112.0 | 38.0 – 112.0 | 63.0 – 101.0 | 59.0 – 124.0 |
|  | Missing | 5 | 27 | 17 | 6 | 24 | 25 |
| **Heart rate (BPM)** |  |  |  |  |  |  |  |
|  | Mean (SD) | 74.8 (12.4) | 79.4 (15.3) | 75.1 (13.5) | 77.4 (12.7) | 80.6 (14.9) | 80.1 (16.3) |
|  | Median [IQR] | 73.0  [66.5 - 83.0] | 78.0  [68.0 – 89.0] | 77.0  [63.0 – 82.0] | 79.0  [69.0 - 86.0] | 80.0  [67.0 – 94.0] | 80.0  [66.0 - 92.0] |
|  | Min - Max | 51.0 – 104.0 | 57.0 – 116.0 | 51 – 112 | 50.0 – 110.0 | 50.0 – 112.0 | 53 – 124 |
|  | Missing | 3 | 26 | 19 | 2 | 22 | 21 |
| **Bloods: Urea & Electrolytes** |  |  |  |  |  |  |  |
| Sodium – normal? | Yes | 17 (33%) | 10 (100%) | 11 (92%) | 14 (30%) | 11 (85%) | 12 (100%) |
|  | No | 0 (-) | 0 (-) | 1 (8%) | 0 (-) | 2 (15%) | 0 (-) |
|  | Not collected | 34 (67%) | 0 (-) | 0 (-) | 32 (70%) | 0 (-) | 0 (-) |
|  | Missing | 0 | 41 | 36 | 2 | 36 | 36 |
| If no, value? | Median [IQR] | - | - | 132 [132 - 132] | - | - | - |
|  | Min - Max | - | - | 132 – 132 | - | - | - |
|  | Missing | - | - | 0 | - | - | - |
| Potassium – normal? | Yes | 17 (33%) | 10 (100%) | 10 (83%) | 13 (28%) | 13 (100%) |  |
|  | No | 0 (-) | 0 (-) | 2 (17%) | 1 (2%) | 0 (-) |  |
|  | Not collected | 34 (67%) | 0 (-) | 0 (-) | 32 (70%) | 0 (-) | 0 (-) |
|  | Missing | 0 | 41 | 36 | 2 | 36 | 36 |
| If no, value? | Median [IQR] | - | - | 3 [3 - 3] | 3.0  [3.0 – 3.0] | - | - |
|  | Min - Max | - | - | 3 - 3 | 3.0 – 3.0 | - | - |
|  | Missing | - | - | 1 | 0 | - | - |
| Chloride – normal? | Yes | 16 (31%) | 10 (100%) | 11 (92%) | 11 (24%) | 12 (100%) | 11 (92%) |
|  | No | 0 (-) | 0 (-) | 1 (8%) | 2 (4%) | 0 (-) | 1 (8%) |
|  | Not collected | 35 (69%) | 0 (-) | 0 (-) | 33 (72%) | 0 (-) | 0 (-) |
|  | Missing | 0 | 41 | 36 | 2 | 37 | 36 |
| If no, value? | Median [IQR] | - | - | 112 [112 - 112] | 101.5  [94.0 – 109.0] | - | 110 [110 - 110] |
|  | Min - Max | - | - | 112 – 112 | 94.0 – 109.0 | - | 110 – 110 |
|  | Missing | - | - | 0 | 0 | - | 0 |
| Creatinine – normal? | Yes | 16 (31%) | 10 (100%) | 11 (92%) | 13 (28%) | 12 (92%) | 12 (100%) |
|  | No | 1 (2%) | 0 (-) | 1 (8%) | 0 (-) | 1 (8%) | 0 (-) |
|  | Not collected | 34 (67%) | 0 (-) | 0 (-) | 33 (72%) | 0 (-) | 0 (-) |
|  | Missing | 0 | 41 | 36 | 2 | 36 | 36 |
| If no, value? | Median [IQR] | 33.0  [33.0 – 33.0] | - | 155 [155 - 155] | - | 42 [42 - 42] | - |
|  | Min - Max | 33.0 – 33.0 | - | 155 – 155 | - | 42 – 42 | - |
|  | Missing | 0 | - | 0 | - | 0 | - |
| eGFR – normal? | Yes | 17 (33%) | 10 (100%) | 11 (92%) | 14 (30%) | 11 (92%) | 11 (100%) |
|  | No | 1 (2%) | 0 (-) | 1 (8%) | 0 (-) | 1 (8%) | 0 (-) |
|  | Not collected | 32 (63%) | 0 (-) | 0 (-) | 32 (70%) | 0 (-) | 0 (-) |
|  | Missing | 1 | 41 | 36 | 2 | 37 | 37 |
| If no, value? | Median [IQR] | - | - | 35 [35 - 35] | - | 58 [58 - 58] | - |
|  | Min - Max | - | - | 35 – 35 | - | 58 – 58 | - |
|  | Missing | 1 | - | 0 | - | 0 | - |
| **Liver Function Tests** |  |  |  |  |  |  |  |
| ALT – normal? | Yes | 15 (29%) | 10 (100%) | 11 (85%) | 12 (26%) | 11 (85%) | 8 (73%) |
|  | No | 2 (4%) | 0 (-) | 2 (15%) | 2 (4%) | 2 (15%) | 3 (27%) |
|  | Not collected | 34 (67%) | 0 (-) | 0 (-) | 32 (68%) | 0 (-) | 0 (-) |
|  | Missing | 0 | 41 | 35 | 1 | 36 | 37 |
| If no, value? | Median [IQR] | 210.0  [109.0 – 311.0] | - | 104.5  [58.0 – 151.0] | 122.5  [54.0 – 191.0] | 123.5  [89.0 – 158.0] | 77  [63 - 137] |
|  | Min - Max | 109.0 – 311.0 | - | 58.0 – 151.0 | 54.0 – 191.0 | 89.0 – 158.0 | 63 – 137 |
|  | Missing | 0 | - | 0 | 0 | 0 | 0 |
| AST – normal? | Yes | 14 (27%) | 10 (100%) | 11 (85%) | 8 (17%) | 8 (73%) | 8 (73%) |
|  | No | 3 (6%) | 0 (-) | 2 (15%) | 5 (11%) | 3 (27%) | 3 (27%) |
|  | Not collected | 34 (67%) | 0 (-) | 0 (-) | 34 (72%) | 0 (-) | 0 (-) |
|  | Missing | 0 | 41 | 35 | 1 | 38 | 37 |
| If no, value? | Median [IQR] | 143.0  [67.0 – 219.5] | - | 225.5  [63.0 – 388.0] | 45.0  [45.0 – 73.0] | 52  [41 – 203] | 86 [63 - 138] |
|  | Min - Max | 67.0 – 219.0 | - | 63.0 – 388.0 | 43.0 – 74.0 | 41 – 203 | 63 - 138 |
|  | Missing | 1 | - | 0 | 0 | 0 | 0 |
| ALP – normal? | Yes | 14 (27%) | 9 (100%) | 11 (85%) | 12 (26%) | 10 (77%) | 11 (100%) |
|  | No | 2 (4%) | 0 (-) | 2 (15%) | 2 (4%) | 3 (23%) | 0 (-) |
|  | Not collected | 35 (69%) | 0 (-) | 0 (-) | 33 (70%) | 0 (-) | 0 (-) |
|  | Missing | 0 | 42 | 35 | 1 | 36 | 37 |
| If no, value? | Median [IQR] | 151.5  [144.0 – 159.0] | - | 159.5  [135.0 – 184.0] | 182.0  [147.0 – 217.0] | 432  [187 - 442] | - |
|  | Min - Max | 144.0 – 159.0 | - | 135.0 – 184.0 | 147.0 – 217.0 | 187 – 442 | - |
|  | Missing | 0 | - | 0 | 0 | 0 | - |
| Albumin – normal? | Yes | 13 (25%) | 9 (90%) | 7 (54%) | 10 (21%) | 8 (62%) | 7 (64%) |
|  | No | 3 (6%) | 1 (10%) | 6 (46%) | 4 (9%) | 5 (38%) | 4 (36%) |
|  | Not collected | 35 (69%) | 0 (-) | 0 (-) | 33 (70%) | 0 (-) | 0 (-) |
|  | Missing | 0 | 41 | 37 | 1 | 36 | 37 |
| If no, value? | Median [IQR] | 27.0  [22.0 – 30.0] | 28  [28 - 28] | 30.5  [27.0 – 32.0] | 33.0  [32.0 – 33.0] | 33  [27 - 34] | 31  [27 - 33] |
|  | Min - Max | 22.0 – 30.0 | 28 – 28 | 23 – 51 | 32.0 – 33.0 | 26 – 34 | 27 - 33 |
|  | Missing | 0 | 0 | 0 | 1 | 0 | 1 |
| **Bloods: Bone profile** |  |  |  |  |  |  |  |
| Calcium – normal? | Yes | 7 (14%) | 5 (71%) | 6 (75%) | 4 (9%) | 5 (63%) | 6 (100%) |
|  | No | 1 (2%) | 2 (29%) | 2 (25%) | 1 (2%) | 3 (38%) | 0 (-) |
|  | Not collected | 43 (84%) | 0 (-) | 0 (-) | 42 (89%) | 0 (-) | 0 (-) |
|  | Missing | 0 | 44 | 40 | 1 | 41 | 42 |
| If no, value? | Median [IQR] | 2.07  [2.07 – 2.07] | - | - | 2.19  [2.19 – 2.19] | - | - |
|  | Min - Max | 2.07 – 2.07 | - | - | 2.19 – 2.19 | - | - |
|  | Missing | 0 | 2 | 2 | 0 | 3 | - |
| Calcium adjusted – normal? | Yes | 8 (16%) | 7 (100%) | 6 (75%) | 5 (11%) | 7 (100%) | 6 (100%) |
|  | No | 0 (-) | 0 (-) | 2 (25%) | 0 (-) | 0 (-) | 0 (-) |
|  | Not collected | 43 (84%) | 0 (-) | 0 (-) | 41 (89%) | 0 (-) | 0 (-) |
|  | Missing | 0 | 44 | 40 | 2 | 42 | 42 |
| If no, value? | Median [IQR] | - | - | - | - | - | - |
|  | Min - Max | - | - | - | - | - | - |
|  | Missing | - | - | 2 | - | - | - |
| Phosphate – normal? | Yes | 8 (16%) | 6 (86%) | 6 (75%) | 4 (9%) | 6 (100%) | 6 (100%) |
|  | No | 0 (-) | 1 (14%) | 2 (25%) | 0 (-) | 0 (-) | 0 (-) |
|  | Not collected | 43 (84%) | 0 (-) | 0 (-) | 43 (91%) | 0 (-) | 0 (-) |
|  | Missing | 0 | 44 | 40 | 1 | 43 | 42 |
| If no, value? | Median [IQR] | - | - | - | - | - | - |
|  | Min - Max | - | - | - | - | - | - |
|  | Missing | - | 1 | 2 | - | - | - |
| CRP – normal? | Yes | 6 (12%) | 4 (67%) | 5 (56%) | 2 (4%) | 2 (40%) | 4 (57%) |
|  | No | 4 (8%) | 2 (33%) | 4 (44%) | 2 (4%) | 3 (60%) | 3 (43%) |
|  | Not collected | 41 (80%) | 0 (-) | 0 (-) | 43 (91%) | 0 (-) | 0 (-) |
|  | Missing | 0 | 45 | 39 | 1 | 44 | 41 |
| If no, value? | Median [IQR] | 51.0  [26.0 – 109.0] | 113  [15 - 211] | 68  [23 – 138.5] | 44.0  [44.0 – 44.0] | 50  [19 - 81] | 14  [11 - 29] |
|  | Min - Max | 11.0 – 176.0 | 15 – 211 | 17 - 170 | 44.0 – 44.0 | 19 – 81 | 11 - 29 |
|  | Missing | 1 | 0 | 0 | 1 | 0 | 0 |
| B12 – normal? | Yes | 5 (10%) | 2 (100%) | 5 (100%) | 0 (-) | 2 (100%) | 3 (100%) |
|  | No | 3 (6%) | 0 (-) | 0 (-) | 0 (-) | 0 (-) | 0 (-) |
|  | Not collected | 43 (84%) | 0 (-) | 0 (-) | 47 (100%) | 0 (-) | 0 (-) |
|  | Missing | 0 | 49 | 43 | 1 | 47 | 45 |
| If no, value? | Median [IQR] | 861.5  [648.0 – 1075.0] | - | - | - | - | - |
|  | Min - Max | 648.0 – 1075.0 | - | - | - | - | - |
|  | Missing | 1 | - | - | - | - | - |
| Full blood count – normal? | Yes | 4 (8%) | 1 (10%) | 4 (29%) | 2 (4%) | 0 (-) | 1 (10%) |
|  | No | 14 (27%) | 9 (90%) | 10 (71%) | 11 (23%) | 12 (100%) | 9 (90%) |
|  | Not collected | 33 (65%) | 0 (-) | 0 (-) | 34 (72%) | 0 (-) | 0 (-) |
|  | Missing | 0 | 41 | 38 | 1 | 37 | 38 |
| Magnesium – normal? | Yes | 4 (8%) | 1 (50%) | 4 (80%) | 1 (2%) | 1 (100%) | 3 (60%) |
|  | No | 1 (2%) | 1 (50%) | 1 (20%) | 0 (-) | 0 (-) | 2 (40%) |
|  | Not collected | 46 (90%) | 0 (-) | 0 (-) | 46 (98%) | 0 (-) | 0 (-) |
|  | Missing | 0 | 49 | 43 | 1 | 48 | 43 |
| If no, value? | Median [IQR] | 1.01  [1.01 – 1.01] | - | - | - | - | - |
|  | Min - Max | 1.01 - 1.01 | - | - | - | - | - |
|  | Missing | 0 | 1 | 1 | - | - | 2 |
| Folate – normal? | Yes | 3 (6%) | 2 (100%) | 2 (50%) | 0 (-) | 0 (-) | 2 (66%) |
|  | No | 6 (12%) | 0 (-) | 2 (50%) | 0 (-) | 2 (100%) | 1 (33%) |
|  | Not collected | 42 (82%) | 0 (-) | 0 (-) | 47 (100%) | 0 (-) | 0 (-) |
|  | Missing | 0 | 49 | 44 | 1 | 47 | 45 |
| If no, value? | Median [IQR] | 2.3  [2.2 – 2.4] | - | - | - | - | - |
|  | Min - Max | 2.2 – 2.4 | - | - | - | - | - |
|  | Missing | 3 | - | 2 | - | 2 | 1 |
| Cholesterol – normal? | Yes | 3 (6%) | 1 (50%) | 1 (50%) | 2 (4%) | 1 (100%) | 1 (50%) |
|  | No | 1 (2%) | 1 (50%) | 1 (50%) | 0 (-) | 0 (-) | 1 (50%) |
|  | Not collected | 47 (92%) | 0 (-) | 0 (-) | 45 (96%) | 0 (-) | 0 (-) |
|  | Missing | 0 | 49 | 46 | 1 | 48 | 46 |
| If no, value? | Median [IQR] | 1, 0.38  [0.38 – 0.38] | 7  [7 - 7] | - | - | - | - |
|  | Min - Max | 0.38 – 0.38 | 7 - 7 | - | - | - | - |
|  | Missing | 0 | 0 | 1 | - | - | 1 |
| PFTs – normal? | Yes | 0 (-) | 0 (-) | 1 (100%) | 0 (-) | 0 (-) | 1 (100%) |
|  | No | 0 (-) | 0 (-) | 0 (-) | 0 (-) | 0 (-) | 0 (-) |
|  | Not collected | 49 (100%) | 0 (-) | 0 (-) | 47 (100%) | 0 (-) | 0 (-) |
|  | Missing | 2 | 51 | 47 | 1 | 49 | 47 |
| **Bloods: Blood borne viruses** |  |  |  |  |  |  |  |
| HIV – normal? | Yes | 10 (20%) | 5 (100%) | 8 (89%) | 13 (28%) | 8 (80%) | 5 (83%) |
|  | No | 1 (2%) | 0 (-) | 1 (11%) | 1 (2%) | 2 (20%) | 1 (17%) |
|  | Not collected | 40 (78%) | 0 (-) | 0 (-) | 33 (70%) | 0 (-) | 0 (-) |
|  | Missing | 0 | 46 | 39 | 1 | 39 | 42 |
| If no, value? | Median [IQR] | - | - | - | 1 | - | - |
|  | Min - Max | - | - | - | - | - | - |
|  | Missing | 1 | - | 1 | - | 2 | 1 |
| Hepatitis B – normal? | Yes | 9 (18%) | 6 (100%) | 8 (89%) | 12 (26%) | 9 (90%) | 6 (100%) |
|  | No | 0 (-) | 0 (-) | 1 (11%) | 0 (-) | 1 (10%) | 0 (-) |
|  | Not collected | 42 (82%) | 0 (-) | 0 (-) | 35 (74%) | 0 (-) | 0 (-) |
|  | Missing | 0 | 45 | 39 | 1 | 39 | 42 |
| If no, value? | Median [IQR] | - | - | - | - | - | - |
|  | Min - Max | - | - | - | - | - | - |
|  | Missing | - | - | 1 | - | 1 | - |
| HCV/Hepatitis C – normal? | Yes | 11 (22%) | 5 (63%) | 6 (60%) | 10 (21%) | 6 (55%) | 3 (50%) |
|  | No | 3 (6%) | 3 (38%) | 4 (40%) | 4 (8%) | 5 (45%) | 3 (50%) |
|  | Not collected | 37 (73%) | 0 (-) | 0 (-) | 32 (67%) | 0 (-) | 0 (-) |
|  | Missing | 0 (-) | 43 | 38 | 1 (2%) | 38 | 42 |
| If no, value? | Median [IQR] | - | - | - | - | - | - |
|  | Min - Max | - | - | - | - | - | - |
|  | Missing | - | 3 | 4 | - | 5 | 3 |
| Sources used to obtain external data | GP records | 44 (86%) | 36 (71%) | 35 (69%) | 39 (81%) | 38 (78%) | 32 (65%) |
|  | NHS Greater Glasgow and Clyde IT services | 25 (49%) | 25 (49%) | 25 (49%) | 25 (52%) | 25 (51%) | 25 (51%) |
|  | Simon community Scotland | 24 (47%) | 21 (41%) | 16 (31%) | 25 (52%) | 24 (49%) | 20 (41%) |
|  | Community Pharmacy Participant Medication Records | 22 (43%) | 0 (-) | 1 (2%) | 22 (46%) | 0 (-) | 0 (-) |
|  | Alcohol and Drug Recovery Services (ADRS) | 1 (2%) | 8 (16%) | 0 (-) | 2 (4%) | 5 (10%) | 0 (-) |
|  | Homelessness support hubs | 0 (-) | 0 (-) | 4 (8%) | 1 (2%) | 1 (2%) | 4 (8%) |
|  | Birmingham and Solihull mental Health Foundations Trust | 0 (-) | 7 (14%) | 15 (29%) | 1 (2%) | 8 (16%) | 14 (29%) |
|  | SIFA Fireside | 0 (-) | 0 (-) | 1 (2%) | 0 (-) | 0 (-) | 0 (-) |

Data are either Mean (SD), Median [IQR] or Number (%) For questions with leading question, percentages reflect proportions within the category.

*Participants were able to select more than one response

**Supplementary material 3: Descriptions of the PHOENIx Intervention**

|  | **PHOENIx Intervention**  **(n=49)** |
| --- | --- |
| **Number of participants having at least one contact from intervention team over 6 months (Face to Face/Telephone)** | |
| **Overall:** |  |
| **Yes** | 42/49 (86%) |
| **No** | 7/49 (14%) |
| **Glasgow:** |  |
| **Yes** | 22/25 (88%) |
| **No** | 3/25 (12%) |
| **Birmingham:** |  |
| **Yes** | 20/24 (83%) |
| **No** | 4/24 (17%) |
| **Proportion of intervention participants having regular weekly contact over 6 months (Face to Face/Telephone)^1^** | |
| **Overall:** |  |
| **Yes** | 18/49 (37%) |
| **No** | 31/49 (63%) |
| **Glasgow:** |  |
| **Yes** | 16/25 (64%) |
| **No** | 9/25 (36%) |
| **Birmingham:** |  |
| **Yes** | 2/22 (8%) |
| **No** | 22/24 (92%) |
| **Number of Face to Face consultations over 6 months per participant^2^** | |
| **Overall:** |  |
| **Mean (SD)** | 10.9 (12.8) |
| **Median (IQR)** | 6.0 [1.0 – 18.0] |
| **Min-Max** | 0 – 64.0 |
| **Glasgow:** |  |
| **Mean (SD)** | 17.4 (14.7) |
| **Median (IQR)** | 18.0 (5.0 – 24.0) |
| **Min-Max** | 0 – 64.0 |
| **Birmingham:** |  |
| **Mean (SD)** | 4.0 (4.6) |
| **Median (IQR)** | 2.5 [0 – 6.0] |
| **Min-Max** | 0 – 18.0 |
| **Number of Telephone consultations per participant over 6 months^2^** | |
| **Overall:** |  |
| **Mean (SD)** | 8.5 (11.9) |
| **Median (IQR)** | 3.0 [0 – 14.0] |
| **Min-Max** | 0 – 46.0 |
| **Glasgow:** |  |
| **Mean (SD)** | 15.0 (13.5) |
| **Median (IQR)** | 13.0 (4.0 – 23.0) |
| **Min-Max** | 0 – 46.0 |
| **Birmingham:** |  |
| **Mean (SD)** | 1.7 (3.2) |
| **Median (IQR)** | 0 [0 – 3.0] |
| **Min-Max** | 0 – 14.0 |

^1^At least 16 weeks over the 6 month period; ^2^Numbers presented are for those with successful contacts only.

**Supplementary material 4: Additional data- Social outcomes**

|  |  | **Usual Care** | | | **PHOENIx Intervention** | | |
| --- | --- | --- | --- | --- | --- | --- | --- |
|  |  | **Baseline**  **(n=51)** | **0-3 Months**  **(n=51)** | **3-6 Months**  **(n=48)** | **Baseline**  **(n=48)** | **0-3 Months**  **(n=49)** | **3-6 Months**  **(n=48)** |
| If the participant has any debt/loan, who Does participant owe debt? * | Phone | 1 (6%) | 0 (-) | 0 (-) | 2 (29%) | 0 (-) | 0 (-) |
|  | Refused to answer | 1 (6%) | 0 (-) | 0 (-) | 2 (29%) | 0 (-) | 0 (-) |
|  | DWP | 1 (6%) | 1 (7%) | 1 (8%) | 2 (29%) | 0 (-) | 0 (-) |
|  | Bills | 1 (6%) | 3 (21%) | 1 (8%) | 1 (14%) | 0 (-) | 0 (-) |
|  | Friends or Family | 0 (-) | 1 (7%) | 0 (-) | 2 (29%) | 2 (20%) | 0 (-) |
|  | Money lender | 1 (6%) | 1 (7%) | 0 (-) | 1 (14%) | 0 (-) | 0 (-) |
|  | Council | 1 (6%) | 0 (-) | 1 (8%) | 0 (-) | 2 (20%) | 0 (-) |
|  | Court | 1 (6%) | 1 (7%) | 0 (-) | 0 (-) | 1 (10%) | 0 (-) |
|  | Drug dealer | 0 (-) | 0 (-) | 0 (-) | 1 (14%) | 0 (-) | 0 (-) |
|  | Benefit fraud | 0 (-) | 0 (-) | 0 (-) | 1 (14%) | 0 (-) | 0 (-) |
|  | Overdraft | 0 (-) | 0 (-) | 0 (-) | 1 (14%) | 0 (-) | 0 (-) |
|  | Rent | 0 (-) | 0 (-) | 0 (-) | 1 (14%) | 0 (-) | 0 (-) |
|  | Universal Credit | 0 (-) | 1 (7%) | 1 (8%) | 0 (-) | 0 (-) | 0 (-) |
|  | Social Security | 0 (-) | 2 (14%) | 2 (15%) | 0 (-) | 2 (20%) | 2 (20%) |
|  | Shop | 0 (-) | 1 (7%) | 0 (-) | 0 (-) | 0 (-) | 0 (-) |
|  | Government | 0 (-) | 1 (7%) | 0 (-) | 0 (-) | 0 (-) | 0 (-) |
|  | Bank | 0 (-) | 1 (7%) | 0 (-) | 0 (-) | 0 (-) | 2 (29%) |
|  | Fine | 0 (-) | 0 (-) | 2 (15%) | 0 (-) | 1 (10%) | 1 (14%) |
|  | RTS | 0 (-) | 1 (7%) | 0 (-) | 0 (-) | 0 (-) | 0 (-) |
|  | Housing benefit | 0 (-) | 0 (-) | 2 (15%) | 0 (-) | 0 (-) | 0 (-) |
|  | Landlord | 0 (-) | 0 (-) | 1 (8%) | 0 (-) | 0 (-) | 0 (-) |
|  | Electronics | 0 (-) | 0 (-) | 1 (8%) | 0 (-) | 0 (-) | 0 (-) |
|  | Credit Cards | 0 (-) | 0 (-) | 0 (-) | 0 (-) | 0 (-) | 1 (14%) |
|  | Researcher decided not to ask/Inappropriate | 0 (-) | 0 (-) | 0 (-) | 0 (-) | 0 (-) | 1 (14%) |
|  | Unknown | 0 (-) | 0 (-) | 0 (-) | 3 (43%) | 0 (-) | 0 (-) |

Data are either Mean (SD), Median [IQR] or Number (%) For questions with leading question, percentages reflect proportions within the category

**Supplementary material 5: Additional data- referrals and additions specific outcomes**

|  |  | **Usual Care** | | | **PHOENIx Intervention** | | |
| --- | --- | --- | --- | --- | --- | --- | --- |
|  |  | **Baseline**  **(n=51)** | **0-3 Months**  **(n=51)** | **3-6 Months**  **(n=48)** | **Baseline**  **(n=48)** | **0-3 Months**  **(n=49)** | **3-6 Months**  **(n=48)** |
| **Referred to: Mental Health Services** |  |  |  |  |  |  |  |
|  | Yes (%) | - | 6 (13%) | 6 (13%) | - | 3 (7%) | 6 (13%) |
|  | Missing | - | 3 | 4 | - | 3 | 2 |
| If yes, no. of times patient referred |  |  |  |  |  |  |  |
|  | Mean (SD) | - | 1 (-) | 1 (-) | - | 1.3 (0.6) | 1.2 (0.4) |
|  | Median (IQR) | - | 1  [1 - 1] | 1  [1 - 1] | - | 1  [1 - 2] | 1  [1 - 1] |
|  | Min-Max | - | 1 – 1 | 1 – 1 | - | 1 – 2 | 1 – 2 |
|  | Missing | - | 0 | 1 | - | 0 | 0 |
| If yes, no. of times patient attended |  |  |  |  |  |  |  |
|  | Mean (SD) | - | 0.8 (0.4) | 0.5 (0.6) | - | 1.3 (0.6) | 0.3 (0.5) |
|  | Median (IQR) | - | 1  [1 - 1] | 0.5  [0 – 1.0] | - | 0  [0 - 0] | 0  [0 – 1.0] |
|  | Min-Max | - | 0 – 1 | 0 – 1 | - | 0 – 0 | 0 – 1 |
|  | Missing | - | 0 | 2 | - | 0 | 0 |
| **Referred to: Rehabilitation** |  |  |  |  |  |  |  |
|  | Yes (%) | - | 1 (2%) | 2 (5%) | - | 3 (7%) | 2 (4%) |
|  | Missing | - | 4 | 4 | - | 3 | 3 |
| If yes, no. of times patient referred |  |  |  |  |  |  |  |
|  | Mean (SD) | - | 1 (-) | 1 (-) | - | 1 (-) | 1 (-) |
|  | Median (IQR) | - | 1  [1 - 1] | 1 [1 - 1] | - | 1  [1 - 1] | 1 [1 - 1] |
|  | Min-Max | - | 1 – 1 | 1 – 1 | - | 1 – 1 | 1 – 1 |
|  | Missing | - | 0 | 0 | - | 0 | 0 |
| If yes, no. of times patient attended |  |  |  |  |  |  |  |
|  | Mean (SD) | - | 1 (-) | 1 (-) | - | 1 (-) | 2 (-) |
|  | Median (IQR) | - | 1  [1 - 1] | 1  [1 - 1] | - | 1  [1 - 1] | 1  [1 - 1] |
|  | Min-Max | - | 1 – 1 | 1 – 1 | - | 1 – 1 | 1 – 1 |
|  | Missing | - | 0 | 1 | - | 0 | 0 |
| **Referred to: Addiction services (e.g. ADRS or CGL)** |  |  |  |  |  |  |  |
|  | Yes (%) | - | 4 (9%) | 8 (19%) | - | 5 (11%) | 11 (24%) |
|  | Missing | - | 4 | 5 | - | 3 | 2 |
| If yes, no. of times patient referred |  |  |  |  |  |  |  |
|  | Mean (SD) | - | 1 (-) | 1 (-) | - | 1 (-) | 1.5 (0.8) |
|  | Median (IQR) | - | 1  [1 - 1] | 1  [1 - 1] | - | 1  [1 - 1] | 1  [1 - 2] |
|  | Min-Max | - | 1 – 1 | 1 – 1 | - | 1 – 1 | 1 – 3 |
|  | Missing | - | 1 | 0 | - | 0 | 0 |
| If yes, no. of times patient attended |  |  |  |  |  |  |  |
|  | Mean (SD) | - | 1 (-) | 0.1 (0.4) | - | 1.3 (1.5) | 0.7 (1.1) |
|  | Median (IQR) | - | 1  [1 - 1] | 0  [0 - 1] | - | 1  [0 - 3] | 0  [0 - 1] |
|  | Min-Max | - | 1 – 1 | 0 – 1 | - | 0 – 3 | 0 – 3 |
|  | Missing | - | 2 | 1 | - | 2 | 1 |
| **Referred to: GP** |  |  |  |  |  |  |  |
|  | Yes (%) | - | 3 (6%) | 5 (11%) | - | 3 (6%) | 2 (4%) |
|  | Missing | - | 4 | 4 | - | 2 | 3 |
| If yes, no. of times patient referred |  |  |  |  |  |  |  |
|  | Mean (SD) | - | 1 (-) | 1.6 (1.3) | - | 1 (-) | 1.0 (-) |
|  | Median (IQR) | - | 1  [1 - 1] | 1  [1 - 1] | - | 1  [1 - 1] | 1  [1 - 1] |
|  | Min-Max | - | 1 – 1 | 1 – 4 | - | 1 – 1 | 1 – 1 |
|  | Missing | - | 0 | 0 | - | 1 | 1 |
| If yes, no. of times patient attended |  |  |  |  |  |  |  |
|  | Mean (SD) | - | 0 (-) | 0.8 (0.4) | - | 0.5 (0.7) | 0.8 (0.4) |
|  | Median (IQR) | - | 0  [0 - 0] | 1  [1 - 1] | - | 0.5  [0 - 1] | 1  [1 - 1] |
|  | Min-Max | - | 0 – 0 | 0 – 1 | - | 0 – 1 | 1 – 1 |
|  | Missing | - | 0 | 0 | - | 1 | 1 |
| **Medicines for problem drug use** |  |  |  |  |  |  |  |
| Methadone | Yes | 34 (87%) | 19 (59%) | 27 (57%) | 32 (94%) | 16 (57%) | 19 (51%) |
|  | No | 5 (13%) | 13 (41%) | 20 (43%) | 2 (6%) | 12 (43%) | 18 (49%) |
|  | Missing | 12 | 19 | 1 | 14 | 21 | 11 |
| If yes, daily dose (mg) | Mean (SD) | 75.5 (27.9) | 78.9 (32.9) | 76.7 (31.2) | 76.1 (30.9) | 84.6 (28.7) | 85.3 (35.4) |
|  | Median [IQR] | 70.0  [50.0 – 90.0] | 80  [45 - 105] | 70 [50 - 100] | 67.5  [57.5 – 100.0] | 75  [60 - 75] | 80 [60 - 110] |
|  | Min - Max | 30.0 – 140.  0 | 30 – 140 | 30 – 140 | 30.0 – 150.  0 | 60 – 150 | 10 – 160 |
|  | Missing | 1 | 0 | 3 | 0 | 0 | 2 |
| Buprenorphine oral/sublingual/with naloxone | Yes | 3 (8%) | 4 (13%) | 3 (6%) | 1 (3%) | 0 (-) | 0 (-) |
|  | No | 36 (92%) | 27 (87%) | 43 (93%) | 33 (97%) | 28 (100%) | 37 (100%) |
|  | Missing | 0 | 20 | 2 | 1 | 21 | 11 |
| If yes, daily dose (mg) | Mean (SD) | 15.3 (1.2) | 14.5 (3.8) | 16.0 (4.0) | 16.0 (-) | - | - |
|  | Median [IQR] | 16.0  [14.0 – 16.0] | 13  [12 - 17] | 16 [12 - 20] | 16.0 [-] | - | - |
|  | Min - Max | 14.0 – 16.0 | 12 – 20 | 12 – 20 | 16.0 – 16.0 | - | - |
|  | Missing | 0 | 0 | 0 | 1 | - | - |
| Burprenorphine injection | Yes | 2 (5%) | 2 (6%) | 2 (4%) | 2 (6%) | 3 (11%) | 2 (5%) |
|  | No | 37 (95%) | 29 (94%) | 43 (96%) | 33 (94%) | 25 (89%) | 35 (95%) |
|  | Missing | 0 | 20 | 3 | 0 | 21 | 11 |
| If yes, how often | Weekly | 0 | 0 (-) | 0 (-) | 0 | 0 (-) | 0 (-) |
|  | Monthly | 2 (4%) | 2 (100%) | 2 (100%) | 2 (4%) | 3 (100%) | 2 (100%) |
| If yes, weekly/ monthly dose (mg) | Mean (SD) | 128.0 (0) | 96.0 (45.3) | 112.0 (22.6) | 112.0 (22.6) | 116.0 (19.6) | 97.0 (1.4) |
|  | Median [IQR] | 128.0  [128.0 - 128.0] | 96  [64 - 128] | 112  [128 - 96] | 112.0  [96.0 - 128.0] | 128  [94 - 128] | 97  [96 - 98] |
|  | Min - Max | 128.0 - 128.0 | 64 – 128 | 96 – 128 | 96.0 - 128.0 | 94 – 128 | 96 – 98 |
|  | Missing | 0 | 0 | 0 | 0 | 0 | 0 |
| Diamorphine | Yes | 0 (-) | 1 (3%) | 0 (-) | 0 (-) | 1 (4%) | 0 (-) |
|  | No | 39 (100%) | 30 (97%) | 44 (100%) | 35 (100%) | 27 (96%) | 34 (100%) |
|  | Missing | 0 | 20 | 4 | 0 | 21 | 14 |
| If yes, daily dose (mg) | Mean (SD) | - | 30 (-) | - | - | 30 (-) | - |
|  | Median [IQR] | - | 30  [30 - 30] | - | - | 30  [30 - 30] | - |
|  | Min - Max | - | 30 – 30 | - | - | 30 – 30 | - |
| Diazepam | Yes | 18 (14%) | 6 (19%) | 6 (13%) | 9 (26%) | 4 (14%) | 9 (23%) |
|  | No | 32 (86%) | 25 (81%) | 40 (87%) | 26 (74%) | 24 (86%) | 30 (77%) |
|  | Missing | 0 | 20 | 2 | 0 | 21 | 9 |
| If yes, daily dose (mg) | Mean (SD) | 26.3 (6.6) | 26.3 (9.0) | 30 (-) | 26.6 (5.0) | 25.0 (5.8) | 25.6 (5.3) |
|  | Median [IQR] | 30.0  [20.0 – 30.0] | 30  [30 - 30] | 30  [30 - 30] | 30.0  [20.0 – 30.0] | 25  [20 - 30] | 30  [20 - 30] |
|  | Min - Max | 14.0 – 30.0 | 8 – 30 | 30 – 30 | 20.0 – 30.0 | 20 – 30 | 20 – 30 |
|  | Missing | 0 | 0 | 0 | 0 | 0 | 0 |
| Number of medicines for problem drug use | Mean (SD) | 1.2 (0.4) | 1.03 (0.7) | 0.9 (0.6) | 1.3 (0.4) | 0.9 (0.7) | 0.9 (0.8) |
|  | Median [IQR] | 1.0  [1.0 – 1.0] | 1  [1 - 1] | 1  [1 - 1] | 1.0  [1.0 – 2.0] | 1  [0 - 1] | 1  [0 - 2] |
|  | Min - Max | 1.0 – 2.0 | 0 – 2 | 0 – 2 | 1.0 – 2.0 | 0 – 2 | 0 – 2 |
|  | Missing | 12 | 21 | 9 | 13 | 21 | 13 |
| 0 | N (%) | 0 (-) | 6 (20%) | 9 (23%) | 0 (-) | 8 (29%) | 13 (37%) |
| 1 | N (%) | 31 (79%) | 17 (57%) | 25 (64%) | 26 (74%) | 15 (54%) | 12 (34%) |
| ≥2 | N (%) | 8 (21%) | 7 (23%) | 5 (13%) | 9 (26%) | 5 (18%) | 10 (29%) |
| **Drug Use: Heroin** |  |  |  |  |  |  |  |
| Frequency (once or more daily/most days) | Once or more daily/most days | 9 (56%) | 3 (38%) | 6 (50%) | 13 (62%) | 6 (75%) | 6 (60%) |
|  | Every few days/weekly | 2 (13%) | 0 (-) | 4 (33%) | 4 (19%) | 1 (13%) | 3 (30%) |
|  | Every two weeks/monthly | 4 (25%) | 1 (13%) | 0 (-) | 3 (14%) | 0 (-) | 1 (10%) |
|  | Rarely | 1 (6%) | 3 (38%) | 2 (17%) | 0 (-) | 1 (13%) | 0 (-) |
|  | Missing | 0 | 1 | 0 | 1 | 0 | 1 |
| Dose | ≤0.4g (≤£10) | 5 (31%) | 3 (38%) | 2 (17%) | 2 (10%) | 1 (13%) | 4 (36%) |
|  | > 0.4g but ≤2g (£11-50) | 7 (44%) | 3 (38%) | 4 (33%) | 12 (57%) | 4 (50%) | 4 (36%) |
|  | >2g but ≤4g (£51-100) | 2 (13%) | 0 (-) | 4 (33%) | 6 (29%) | 3 (38%) | 1 (9%) |
|  | Refused to answer | 1 (6%) | 1 (13%) | 2 | 0 (-) | 0 (-) | 2 |
|  | Missing | 1 | 1 | 9 (75%) | 1 | 0 | 6 (54%) |
| Route | Intravenous | 11 (69%) | 4 (50%) | 0 (-) | 14 (67%) | 5 (63%) | 0 (-) |
|  | Snort | 0 (-) | 0 (-) | 3 (25%) | 0 (-) | 0 (-) | 4 (36%) |
|  | Smoke | 5 (31%) | 2 (25%) |  | 7 (33%) | 3 (38%) |  |
|  | Refused to answer | 0 (-) | 1 (13%) | 0 (-) | 0 (-) | 0 (-) | 0 (-) |
|  | Missing | 0 | 1 | 0 | 0 | 0 | 1 |
| **Drug use: Cocaine** |  |  |  |  |  |  |  |
| Frequency (once or more daily/most days) | Once or more daily/most days | 0 (-) | 6 (43%) | 4 (22%) | 0 (-) | 4 (33%) | 8 (50%) |
|  | Every few days/weekly | 8 (33%) | 4 (29%) | 6 (33%) | 14 (54%) | 6 (50%) | 6 (38%) |
|  | Every two weeks/monthly | 5 (21%) | 1 (7%) | 5 (28%) | 4 (15%) | 1 (8%) | 1 (6%) |
|  | Rarely | 2 (8%) | 2 (14%) | 3 (17%) | 1 (4%) | 0 (-) | 0 (-) |
|  | Refused to answer | 1 (4%) | 0 (-) | 0 (-) | 0 (-) | 0 (-) | 0 (-) |
|  | Missing | 1 | 1 | 0 | 0 | 1 | 1 |
| Dose | ≤1 bag (0.4g, 2 lines, ≤£10) | 7 (29%) | 4 (29%) | 1 (6%) | 1 (4%) | 1 (8%) | 3 (19%) |
|  | > 1 bag - 2 bags (£10-20) | 6 (25%) | 2 (33%) | 3 (17%) | 8 (31%) | 4 (33%) | 7 (44%) |
|  | > 2 bags - 1g (£21-25) | 0 (-) | 2 (14%) | 1 (6%) | 4 (15%) | 3 (25%) | 0 (-) |
|  | >1g (2.5 bags, >£25) | 10 (42%) | 5 (36%) | 10 (56%) | 13 (50%) | 3 (25%) | 4 (25%) |
|  | Refused to answer | 1 (4%) | 0 (-) | 0 (-) | 0 (-) | 0 (-) | 0 (-) |
|  | Missing | 0 | 1 | 3 | 0 | 1 | 2 |
| Route | Intravenous | 14 (58%) | 6 (43%) | 11 (61%) | 15 (58%) | 6 (50%) | 5 (31%) |
|  | Smoke | 8 (33%) | 5 (36%) | 4 (22%) | 10 (38%) | 5 (42%) | 8 (50%) |
|  | Snort | 2 (8%) | 1 (7%) | 2 (11%) | 1 (4%) | 1 (8%) | 3 (19%) |
|  | Missing | 0 | 2 | 1 | 0 | 0 | 0 |
| **Drug use: Street Valium** |  |  |  |  |  |  |  |
| Frequency (once or more daily/most days) | Once or more daily/most days | 3 (30%) | 6 (75%) | 7 (58%) | 0 (-) | 7 (88%) | 6 (86%) |
|  | Every few days/weekly | 4 (40%) | 1 (13%) | 3 (25%) | 3 (75%) | 1 (13%) | 1 (14%) |
|  | Every two weeks/monthly | 1 (10%) | 0 (-) | 1 (8%) | 0 (-) | 0 (-) | 0 (-) |
|  | Rarely | 2 (20%) | 1 (13%) | 1 (8%) | 1 (25%) | 0 (-) | 0 (-) |
| Dose | 1-10 tablets | 4 (40%) | 2 (25%) | 4 (33%) | 3 (75%) | 3 (38%) | 3 (43%) |
|  | 11-25 tablets | 2 (20%) | 2 (25%) | 5 (42%) | 0 (-) | 4 (50%) | 3 (43%) |
|  | 26-50 tablets | 2 (20%) | 3 (38%) | 0 (-) | 1 (25%) | 1 (13%) | 0 (-) |
|  | 51-100 tablets | 2 (20%) | 1 (13%) | 2 (17%) | 0 (-) | 0 (-) | 0 (-) |
|  | >100 tablets | 0 (-) | 0 (-) | 0 (-) | 0 (-) | 0 (-) | 0 (-) |
|  | Missing | 0 | 0 | 1 | 0 | 0 | 1 |
| **Drug use: Spice** |  |  |  |  |  |  |  |
| Only used in prison | Yes | 0 (-) | 0 (-) | 1 (33%) | 0 (-) | 0 (-) | 0 (-) |
|  | Missing | 0 | 0 | 0 | 0 | 0 | 0 |
| Frequency (once or more daily/most days) | Once or more daily/most days | 2 (67%) | 3 (75%) | 4 (100%) | 4 (80%) | 2 (100%) | 1 (33%) |
|  | Every few days/weekly | 0 (-) | 0 (-) | 0 (-) | 1 (20%) | 0 (-) | 1 (33%) |
|  | Every two weeks/monthly | 1 (33%) | 0 (-) | 0 (-) | 0 (-) | 0 (-) | 0 (-) |
|  | Rarely | 0 (-) | 1 (25%) | 0 (-) | 0 (-) | 0 (-) | 1 (33%) |
| **Drug use: Street Gabapentoids** |  |  |  |  |  |  |  |
| Frequency (once or more daily/most days) | Once or more daily/most days | 4 (67%) | 0 (-) | 1 (25%) | 1 (25%) | 2 (50%) | 0 (-) |
|  | Every few days/weekly | 0 (-) | 1 (50%) | 3 (75%) | 2 (50%) | 1 (25%) | 2 (100%) |
|  | Every two weeks/monthly | 0 (-) | 0 (-) | 0 (-) | 1 (25%) | 0 (-) | 0 (-) |
|  | Rarely | 1 (17%) | 1 (50%) | 0 (-) | 0 (-) | 1 (25%) | 0 (-) |
|  | Refused to answer | 1 (17%) | 0 (-) | 0 (-) | 0 (-) | 0 (-) | 0 (-) |
| **Drug use: cannabis** |  |  |  |  |  |  |  |
| Frequency (once or more daily/most days) | Once or more daily/most days | 6 (55%) | 6 (75%) | 6 (50%) | 10 (50%) | 7 (58%) |  |
|  | Every few days/weekly | 2 (18%) | 1 (13%) | 1 (8%) | 6 (30%) | 3 (25%) | 4 (50%) |
|  | Every two weeks/monthly | 0 (-) | 1 (13%) | 1 (8%) | 1 (5%) | 1 (8%) | 3 (38%) |
|  | Rarely | 2 (18%) | 0 (-) | 3 (25%) | 3 (15%) | 1 (8%) | 0 (-) |
|  | Refused to answer | 1 (9%) | 0 (-) | 0 (-) | 0 (-) | 0 (-) | 1 (13%) |

**Participants who reported ‘YES’ to current heroin use at Baseline and ‘NO’ at 6 month follow up**

|  |  | **Usual Care**  **(n=51)** | **PHOENIx Intervention**  **(n=48)** | **TOTAL**  **(n=99)** |
| --- | --- | --- | --- | --- |
| Current heroin use  Baseline = Yes  then 6M = No | N | 4 (8%) | 4 (8%) | 1. (8%) |

Of the eight participants above, the following table evaluates the number of participants who were NOT prescribed any of the opioid substitution therapy (‘Methadone’/’Buprenorphine oral/sublingual/with naloxone’/’Burprenorphine injection’/’Diamorphine’) at baseline but were prescribed at 6 months follow up.

|  |  | **Usual**  **Care** | **PHOENIx Intervention** | **TOTAL** |
| --- | --- | --- | --- | --- |
|  | | | | |
| Prescribed at least any one of the four prescription items at  Baseline = No then 6M = Yes | N | 0/4 | 0/4 | 0/8 |
|  | | | | |
| Methadone  Baseline = No then 6M = Yes | N | 1/4 | 0/4 | 1/8 |
| Buprenorphine oral/sublingual/with naloxone  Baseline = No then 6M = Yes | N | 0/4 | 0/4 | 0/8 |
| Buprenorphine injection  Baseline = No then 6M = Yes | N | 0/4 | 1/4 | 1/8 |
| Diamorphine  Baseline = No then 6M = Yes | N | 0/4 | 0/4 | 0/8 |
